# Light-Promoted Lysosomal Escape of a Phthalocyanine and Antisense Oligonucleotide-Complexed G-Quadruplex for Dual Photodynamic and Antisense Therapy

**DOI:** 10.1021/acsptsci.4c00384

**Published:** 2024-09-25

**Authors:** Dick Yan Tam, Wendy K. M. Lau, Yosephine Tania Limanto, Dennis K. P. Ng

**Affiliations:** Department of Chemistry, The Chinese University of Hong Kong, Shatin, N.T., Hong Kong 999077, China

**Keywords:** antisense therapy, G-quadruplex, lysosomal escape, phthalocyanine, photodynamic therapy

## Abstract

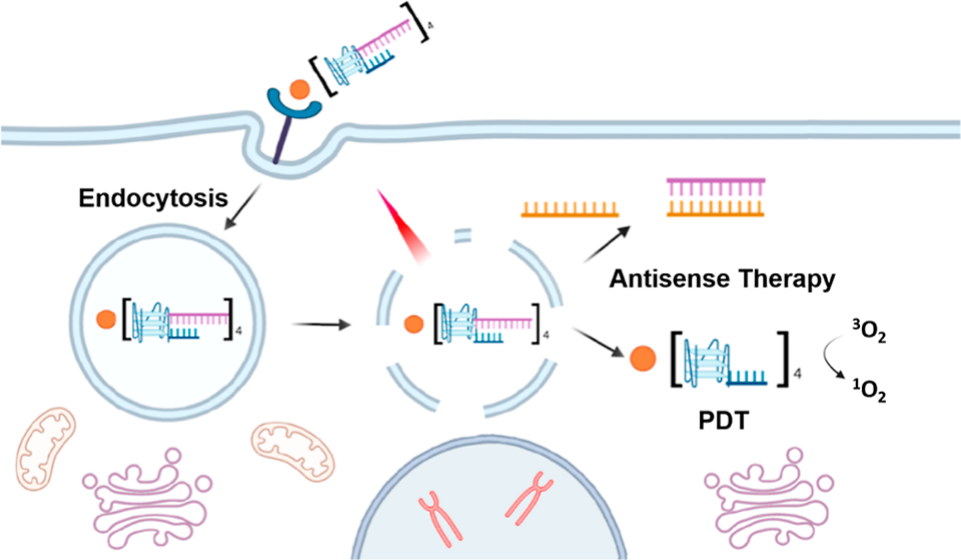

Combination therapy has been proven as an effective strategy for cancer treatment. To this end, we report herein a self-assembled nucleic acid–based complex for dual photodynamic and antisense therapy. It contains a nucleolin-targeting As1411-based G-quadruplex platform, a partially hybridized antisense oligonucleotide 4625, which can inhibit the antiapoptotic protein B cell lymphoma-xL inducing apoptotic cell death, and a zinc(II) phthalocyanine (ZnPc)-based photosensitizer held by noncovalent interactions. Through a series of in vitro experiments, we have demonstrated that this DNA complex can be internalized selectively to nucleolin-overexpressed MCF-7 and A549 cells through receptor-mediated endocytosis and is localized in the lysosomes. Upon light irradiation, the photosensitization of ZnPc triggers the formation of reactive oxygen species for cell killing and promotes the lysosomal escape of 4625 for antisense therapy. The combined therapeutic effect can eliminate the cancer cells effectively with a half maximal inhibitory concentration of ca. 0.5 μM.

## Introduction

Antisense therapy has emerged as a promising anticancer modality.^1−3^ It utilizes a short oligonucleotide (ON) (usually 17–22 nucleotides in length) designed to be complementary to a selected gene’s mRNA that can specifically inhibit the production of the corresponding functional protein to attain a therapeutic effect. This approach exhibits high specificity, which is stemmed from the high fidelity of Watson–Crick hybridization and the estimate that a particular sequence of 17–22 bases in DNA occurs only once within the human genome. Compared with the traditional anticancer therapies, antisense therapy is relatively noninvasive and results in minimal side effects.^4^ It can also be applied repeatedly without noticeable drug resistance due to its unique and well-defined therapeutic mechanism. However, the barriers in internalization and lysosomal escape of antisense ONs affect their drug action and restrict the clinical use of this modality.^5^ In an attempt to resolve these problems, a wide range of cationic polymers and lipids have been explored as carriers for transfection.^6,7^ Through electrostatic interactions, these cationic carriers can compress the negatively charged antisense ONs to facilitate the delivery into cells. The associated proton sponge effect can also promote the lysosomal escape,^8^ favoring the subsequent silencing of the target gene expression. Unfortunately, the intrinsic toxicity of these highly cationic materials inevitably causes severe side effects and hampers the clinical translation. As a result, there is a strong demand of advanced nonviral ON carriers for effective and safe transfection.^9−12^

G-quadruplexes contain a guanine-rich ON sequence that can form a self-assembled G-quartet with four Hoogsteen-paired and coplanar guanines, serving as structural and functional motifs for diverse applications.^13^ Recent studies have shown that they can be used to deliver functional nucleic acids without the need of cationic carriers.^14−16^ Some sequences can also serve as aptamers for binding a specific biomarker of cancer cells.^17,18^ As1411, for example, is one of the most studied and promising candidates, which can recognize the external domain of nucleolin, a phosphoprotein overexpressed on the membrane of cancer cells.^19^ However, as DNA complexes are generally internalized through receptor-mediated endocytosis, they are usually trapped in endo/lysosomes, where the nucleic acids are prone to be degraded by the abundant enzymes therein, compromising the gene therapeutic efficacy. To address this issue, photosensitizers have been used to disrupt the endo/lysosomal membranes to promote the escape of antisense ON-containing complexes by the reactive oxygen species (ROS) generated upon light irradiation.^20−23^ Apart from this photochemical internalization process, the ROS can also cause cytotoxicity, and the resulting photodynamic therapy (PDT) can eliminate cancer cells with antisense therapy in a synergistic manner, boosting the anticancer efficacy.^21−27^

For most of these complexes, photosensitizers are loaded into nanoparticles with antisense ONs.^23−25,27^ Alternatively, photosensitizers are first conjugated with these nucleic acids, and then the resulting conjugates are loaded on a nanoplatform.^21,26^ We report herein a new design using a self-assembled G-quadruplex platform to carry a photosensitizer and an antisense ON. With an aptamer-behaved secondary structure, the resulting complex can be internalized selectively into cancer cells. It can also promote the lysosomal escape of the antisense ON through photochemical internalization, inducing an antisense therapeutic effect that can synergize the photodynamic action of the photosensitizer. These cell-selective and synergistic cytotoxic effects have been demonstrated through a series of in vitro experiments using a range of cell lines. To the best of our knowledge, integration of several functional components in a single DNA entity for therapeutic application has rarely been reported.^14−16^

## Results and Discussion

### Design, Preparation, and Characterization

To assemble this DNA complex, the aforementioned nucleolin-targeting As1411 sequence (5′-GGTGGTGGTGGTTGTGGTGGTGGTGG-3′) was used as the backbone, which was appended with a short sequence (5′-AACGGAGGCTGGGAT-3′) that is partially complementary to the antisense ON 4625 (see Table S1 in Supporting Information for the sequences of all these ONs). This antisense ON is known to exhibit a strong bispecific activity toward B cell lymphoma-2 (Bcl-2) and B cell lymphoma-xL (Bcl-xL) on the transcript and protein levels, inducing apoptosis in cancer cells.^28^ The former antiapoptotic protein has also been found to inhibit most types of apoptotic cell death through regulating an antioxidant pathway.^29^ Hence, it was expected that effective release of 4625 to the cytosol of cancer cells can efficiently downregulate these proteins, causing cell death by apoptosis and promoting the PDT effect in a synergistic manner.

**Scheme 1:**
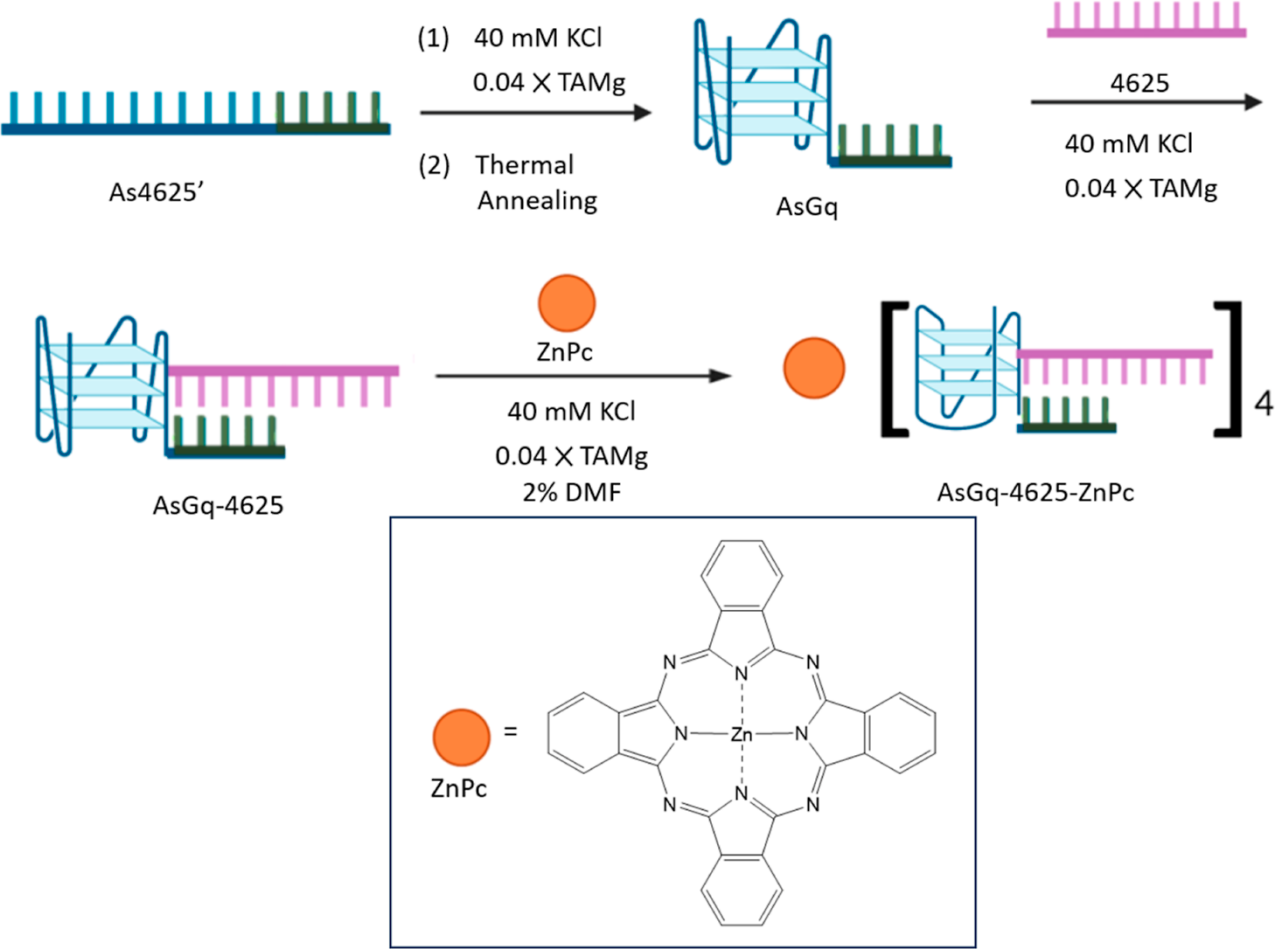
Synthetic Route of **AsGq-4625-ZnPc**

**Figure 1:**
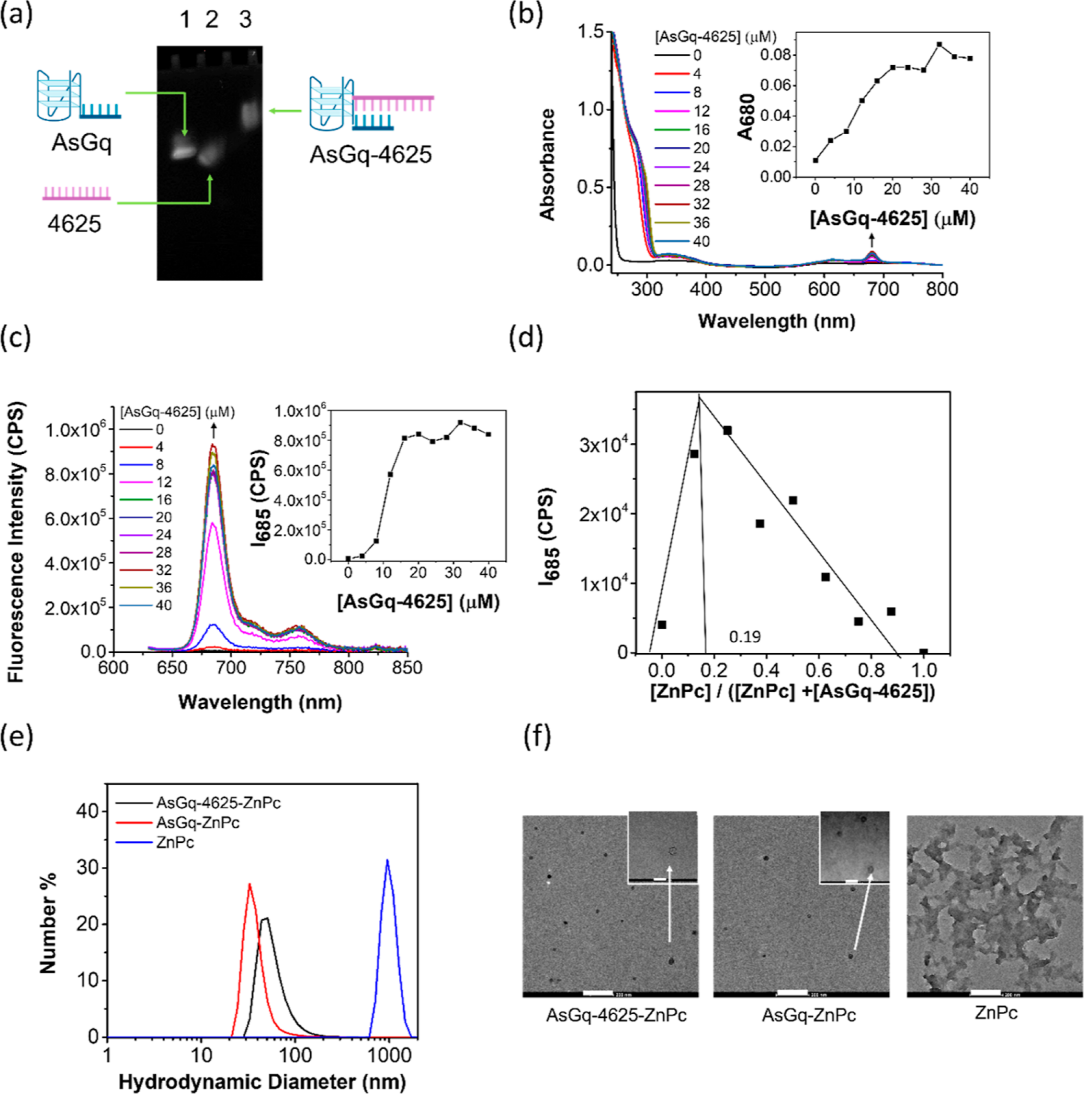
(a) 6.5% Native PAGE supporting the formation of **AsGq-4625.** Change in the (b) electronic absorption and (c) fluorescence (λ_ex_ = 610 nm) spectra of ZnPc (4 μM) upon addition of **AsGq-4625** (up to 40 μM) in TAMg buffer (0.04×) with 40 mM KCl and 2% DMF (v/v). The insets show the variation of the absorbance at 680 nm or fluorescence intensity at 685 nm with the concentration of **AsGq-4625**. (d) Job’s plot analysis of the fluorescence data for the titration of ZnPc with **AsGq-4625**. The total concentration of ZnPc and **AsGq-4625** was fixed at 4 μM. (e) Hydrodynamic diameter distribution of **AsGq-4625-ZnPc**, **AsGq-ZnPc**, and ZnPc in TAMg buffer (0.04×) with 40 mM KCl and 2% DMF (v/v) determined by DLS. (f) TEM images of **AsGq-4625-ZnPc**, **AsGq-ZnPc**, and ZnPc. Scale bar: 200 or 50 (for the insets) nm.

As shown in Scheme 1, the overall sequence labeled as As4625’ (Table S1) underwent self-assembly in a TAMg buffer solution (0.04×) with 40 mM KCl, forming a G-quadruplex labeled as **AsGq** after annealing at 95 °C for 5 min followed by slow cooling to 4 °C. The circular dichroism (CD) spectrum of **AsGq** in this solution at ambient temperature (Figure S1) showed extrema at 262 (+) and 238 (−) nm, indicating that the G-quadruplex formed adopts a parallel structure.^30^ This secondary structure was then hybridized with the antisense ON 4625 (Table S1) also in TAMg buffer (0.04×) with 40 mM KCl to give **AsGq-4625**. The formation of this hybridized product was supported by native polyacrylamide gel electrophoresis (PAGE), which showed a new band with a lower mobility compared with the bands of **AsGq** and 4625 (Figure 1a).

A zinc(II) phthalocyanine (ZnPc)-based photosensitizer was then introduced to this DNA complex. Owing to the strong absorption in the far-red region (λ_abs_ ≈ 670 nm, ε ≈ 10^5^ M^–1^ cm^–1^), high singlet oxygen generation efficiency, and excellent photostability, ZnPc derivatives behave as superior photosensitizers for PDT.^31^ The large hydrophobic π system can also promote the stacking on G-quadruplexes through π–π interactions.^32^ The complexation of several ZnPc derivatives and As1411-based G-quadruplexes has been studied, including the effects on the stability of the secondary structures and the photodynamic activity of the photosensitizers.^33^ To study the binding interactions between ZnPc and the G-quadruplex structure, the change in electronic absorption and fluorescence spectra of ZnPc (4 μM) upon addition of various concentrations of **AsGq** (up to 40 μM) in TAMg buffer (0.04×) with 40 mM KCl and 2% *N*,*N*-dimethylformamide (DMF) (v/v) was monitored. As shown in Figure S2a,b, the initially vanished Q-band absorption at ca. 680 nm became slightly visible, and the intensity of the fluorescence band at 685 nm was largely restored with increasing the concentration of **AsGq**. These results indicated that the G-quadruplex could disrupt the aggregation of ZnPc in this aqueous medium. Very similar results were obtained for the titration study using the 4625-hybridized **AsGq-4625** (Figure 1b,c). Job’s plot analysis of the fluorescence data revealed a 4:1 (G-quadruplex/ZnPc) binding stoichiometry for both **AsGq** (Figure S2c) and **AsGq-4625** (Figure 1d), indicating that on average four G-quadruplex strands are required to load one molecule of ZnPc, which was also observed by Park et al. for their G-quadruplex-decorated Au nanomachines.^34^ The corresponding binding constants were estimated to be (1.6 ± 0.5) × 10^4^ and (1.4 ± 0.5) × 10^4^ M^–4^, respectively, by fitting the concentration-dependent fluorescence data with a modified Benesi–Hildebrand equation as reported previously.^35^ It has been found that the binding properties between G-quadruplexes and phthalocyanines depend largely on the substitution patterns, substituents, and the metal center of phthalocyanines.^32^ The relatively low binding constants suggested that the unsubstituted ZnPc binds weakly to the As1411-based G-quadruplex. It might also explain the different binding stoichiometry of these complexes and those containing cationic phthalocyanine derivatives, some of which have been reported to bind G-quadruplexes in a 1:1 or 2:1 manner.^32,36^

Based on these results, a solution of ZnPc in DMF (0.2 mM, 2 μL) was then mixed with a solution of **AsGq-4625** in TAMg buffer (0.04×) (16 μM, 0.1 mL) with 40 mM KCl to give the target therapeutic agent **AsGq-4625-ZnPc** (Scheme 1). Under these conditions, the number-averaged hydrodynamic diameter of this complex was determined to be 58.2 ± 24.5 nm with a polydispersity index (PDI) of 0.265 by dynamic light scattering (DLS) (Figure 1e). The size was slightly larger than that of the non-4625-conjugated analogue **AsGq-ZnPc** (37.9 ± 14.3 nm; PDI = 0.264) formed by mixing **AsGq** with ZnPc under the same conditions. The nanoscale of these complexes suggested that the 4:1 binding stoichiometry was just the relative amounts of the two components in the complexes. For ZnPc, the size was much larger (1085 ± 75 nm; PDI = 0.349), indicating that it was highly aggregated in this aqueous medium, and the aggregation could be effectively disrupted by the G-quadruplex.

The morphology of these nanoparticles was further examined by transmission electron microscopy (TEM). As shown in Figure 1f, the nanoparticles of **AsGq-4625-ZnPc** and **AsGq-ZnPc** were nearly spherical in shape with a diameter of 36.7 ± 8.7 and 25.6 ± 6.1 nm, respectively. The values were comparable with those determined by DLS. The apparently smaller size measured by TEM can be attributed to the shrinkage of the samples upon drying before the measurements. In contrast, ZnPc formed a network on the TEM grid, for which the diameter could not be determined. This observation further confirmed that the As1411-based G-quadruplex can disstack ZnPc, and the hybridization of 4625 does not significantly affect the complexation.

### Stability of the G-Quadruplexes

**Figure 2:**
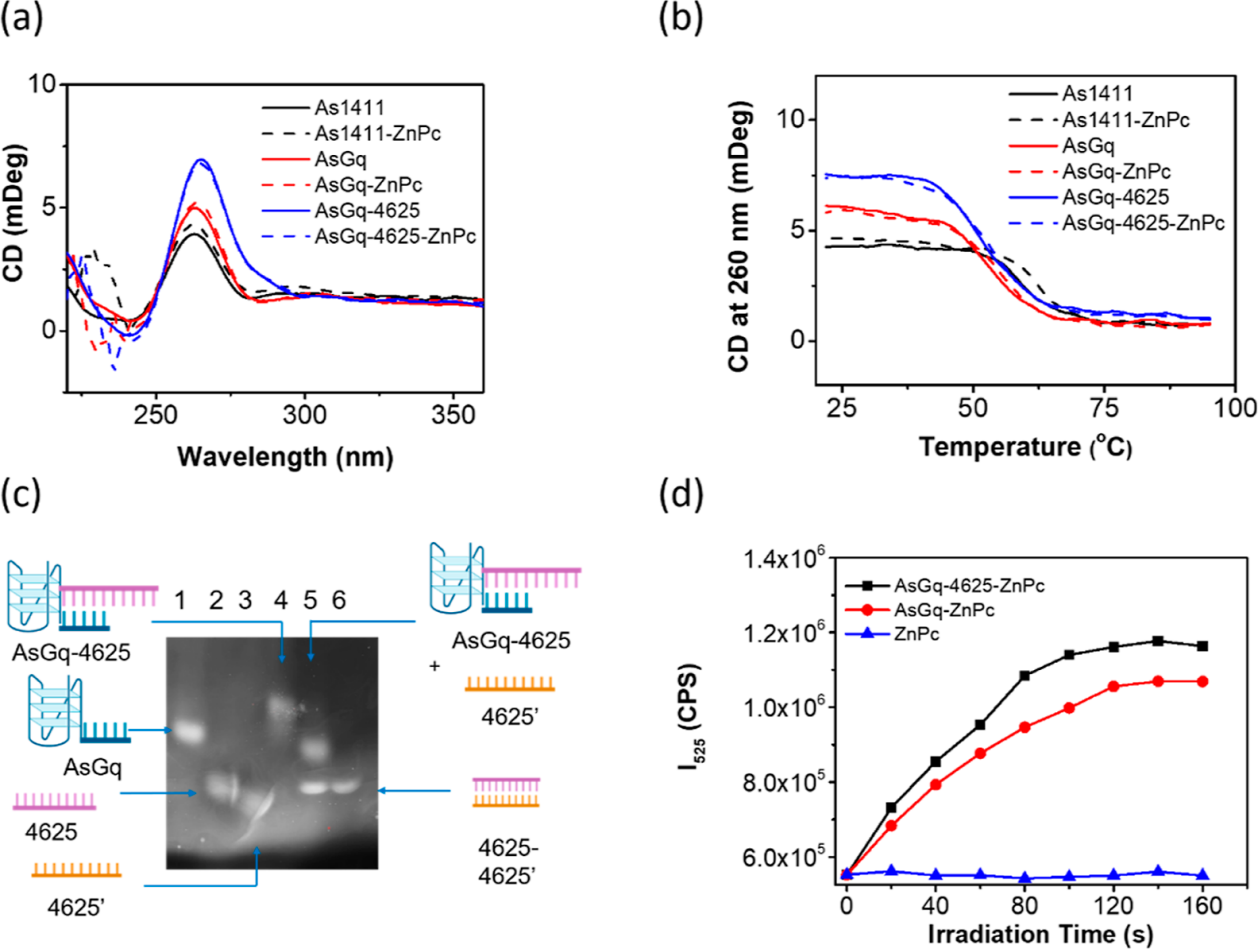
(a) CD spectra of As1411, **AsGq**, and **AsGq-4625** (16 μM) in TAMg buffer (0.04×) with 40 mM KCl and 2% DMF (v/v) with or without the addition of ZnPc (4 μM). (b) The corresponding CD melting curves by monitoring the change in the amplitude at 260 nm over the temperature range of 22–95 °C. (c) 8% Native PAGE supporting the strand displacement of 4625 from **AsGq-4625** by 4625′. (d) Change in the fluorescence intensity of SOSG at 525 nm along with the irradiation time using **AsGq-ZnPc**, **AsGq-4625-ZnPc**, and ZnPc as the photosensitizers in the above aqueous medium with light irradiation (λ > 610 nm).

To confirm that the G-quadruplex structure was not altered after binding with ZnPc, the CD spectra of the G-quadruplexes formed by As1411, **AsGq**, and **AsGq-4625**, respectively, were recorded and compared with or without the addition of ZnPc so that the effects of the short appended-ON and the antisense ON 4625 could also be investigated. Similar to the CD spectrum of **AsGq** (Figure S1), the spectra of the other two structures also showed extrema at 262 (+) and 238 (−) nm, and the addition of ZnPc did not cause a significant change (Figure 2a), showing that the parallel G-quadruplex structure remained intact after the addition of 4625 and ZnPc.

The thermal stability of these structures was then studied by monitoring their denaturation upon heating with CD. The G-quadruplexes of As1411, **AsGq**, and **AsGq-4625** were first generated upon the annealing treatment as described above with or without the addition of ZnPc, and then reversible CD measurements were performed. Figure 2b shows the corresponding CD melting curves by monitoring the change in the amplitude at 260 nm over the temperature range of 22–95 °C. The melting temperatures (*T*_m_) were found to be 59.8, 53.0, and 52.3 °C for the G-quadruplexes of As1411, **AsGq**, and **AsGq-4625**, respectively. Upon complexation with ZnPc, the values slightly increased to 61.7, 54.6, and 53.1 °C, respectively. These results indicated that ZnPc could slightly enhance the thermal stability of the G-quadruplex structure. Lengthening of the strand of As1411 and further hybridization with 4625, however, induced an opposite effect.

To examine the biostability, **AsGq-ZnPc** and **AsGq-4625-ZnPc** were incubated in Dulbecco’s modified Eagle’s medium (DMEM) with 10% (v/v) fetal bovine serum (FBS) at 37 °C over a period of 24 h. The complexes were denatured with formamide at different time points, and the resulting mixtures were analyzed using 15% denaturing PAGE followed by staining with Shinny green and quantification of the band intensity with ImageJ. As shown in Figure S3a, **AsGq-ZnPc** exhibited slow degradation upon FBS digestion with a half-life of 4.5 h. In contrast, **AsGq-4625-ZnPc** was stable under these conditions as shown by the fingerprint band pattern of each of the two DNA components without additional bands appeared (Figure S3b). The fluorescence intensity remained essentially unchanged for both complexes over this period of time (Figure S3c,d), showing that the G-quadruplex structure remained intact in this cell culture medium for at least 24 h. The denaturation of **AsGq-ZnPc** might be due to the appended ON segment, which could be stabilized by the hybridization with 4625, of which the biostability was enhanced by the 2′-OMe and phosphorothioate modifications.

### Strand Displacement

To study the displacement of 4625 by target mRNA, native PAGE analysis was performed, using 4625’ (Table S1), the fully complementary sequence of 4625, as an analogue of the target Bcl-2 and Bcl-xL specific mRNA. In Figure 2c, the bands of **AsGq**, 4625, and 4625′ are shown in lanes 1, 2, and 3, respectively, as references. For **AsGq-4625**, a single band with the lowest mobility was observed in the gel (lane 4). Upon addition of 4625′, two new bands with a higher mobility appeared (lane 5), of which the positions matched with those of **AsGq** (lane 1) and the hybrid of 4625 and 4625′ (lane 6). These results suggested that the antisense ON 4625 could be displaced by target mRNA.

### Singlet Oxygen Generation

With a ZnPc-based photosensitizing unit, **AsGq-ZnPc** and **AsGq-4625-ZnPc** were expected to be able to generate singlet oxygen upon light irradiation. By using singlet oxygen sensor green (SOSG) as a singlet oxygen probe, the photosensitizing property of these two assemblies was studied. As shown in Figure 2d, both of them could remarkably trigger the fluorescence emission of SOSG at 525 nm along with the irradiation time, showing that they could generate singlet oxygen effectively. In contrast, owing to the strong aggregation tendency leading to self-quenching, ZnPc could not behave as a photosensitizer in the aqueous medium. However, complexation with a G-quadruplex structure could effectively perturb the stacking tendency and restore its singlet oxygen generation efficiency.

To examine whether the singlet oxygen generated would attack the ONs as reported previously^37^ and degrade the G-quadruplex structure, solutions of **AsGq-ZnPc** and **AsGq-4625-ZnPc** in DMEM were irradiated with red light (λ > 610 nm) with a fluence rate of 23 mW cm^–2^ for 10 min, giving a total fluence of 13.8 J cm^–2^, which was the light dose used for the subsequent study of the photocytotoxicity (see below). After concentration, the samples were analyzed using 15% denaturing PAGE. The results are shown in Figure S4, which also displays the results in the absence of light irradiation for comparison. It can be seen that both the position and the intensity of the bands were not significantly changed upon light irradiation for both complexes, which suggested that they remained intact under these conditions.

### Cellular Uptake

The internalization of **AsGq-4625-ZnPc** was monitored using confocal microscopy and flow cytometry. The cell-selective property of this DNA complex was demonstrated using the nucleolin-positive MCF-7 human breast carcinoma cells^38^ and A549 human lung carcinoma cells,^39^ as well as the nucleolin-negative L929 mouse fibroblast cells.^40^ As shown in Figure S5a, a noticeable fluorescence signal could be observed inside the MCF-7 cells after incubation with **AsGq-4625-ZnPc** ([ZnPc] = 0.5 μM) for 4 h, and the intensity increased significantly when a higher concentration of the complex ([ZnPc] up to 2 μM) was used. The concentration of ZnPc refers to the feedstock concentration used, assuming that all the ZnPc molecules were complexed with the G-quadruplex. Figure S5b shows the corresponding quantified intracellular fluorescence intensities, in which the remarkable increase in fluorescence intensity can be easily seen. With a fixed concentration of **AsGq-4625-ZnPc** ([ZnPc] = 2 μM), the fluorescence intensity was also found to increase significantly with the incubation time as shown in Figure S5c,d. These results showed that **AsGq-4625-ZnPc** could be internalized readily into these nucleolin-positive cells.

**Figure 3:**
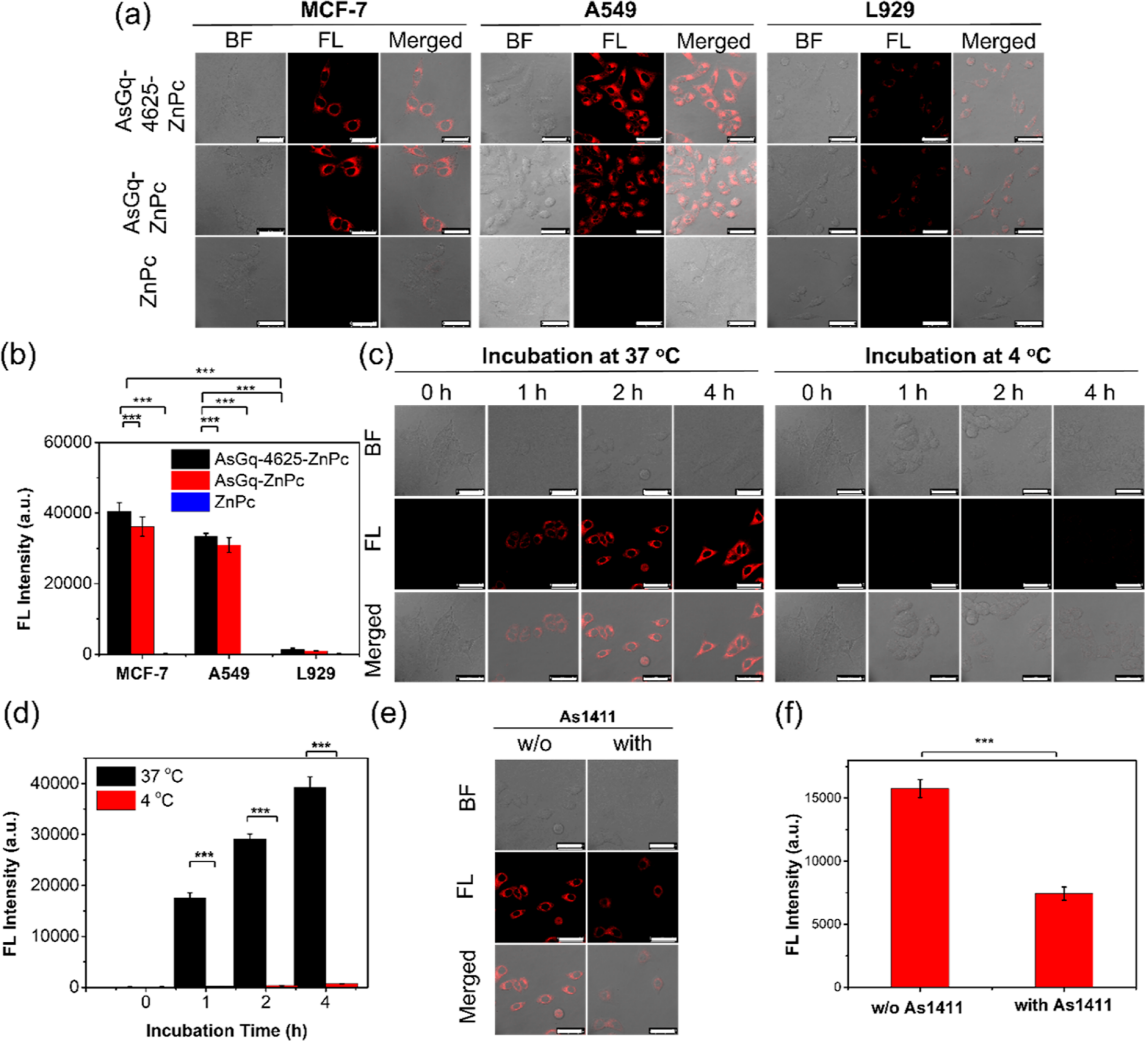
(a) Bright field, fluorescence, and the merged confocal images of MCF-7, A549, and L929 cells after incubation with **AsGq-4625-ZnPc**, **AsGq-ZnPc**, or ZnPc ([ZnPc] = 2 μM) in a serum-free medium for 4 h. Scale bar: 50 μm. (b) Mean intracellular fluorescence intensities under the conditions specified in (a) determined by flow cytometry. (c) Bright field, fluorescence, and the merged confocal images of MCF-7 cells after incubation with **AsGq-4625-ZnPc** ([ZnPc] = 2 μM) in a serum-free medium for different periods of time at 37 and 4 °C. Scale bar: 50 μm. (d) Mean intracellular fluorescence intensities under the conditions specified in (c) determined by flow cytometry. (e) Bright field, fluorescence, and the merged confocal images of MCF-7 cells with or without preincubation with free G-quadruplex of As1411 (8 μM) for 30 min, followed by incubation with **AsGq-4625-ZnPc** ([ZnPc] = 2 μM) in a serum-free medium for 2 h at 37 °C. (f) Mean intracellular fluorescence intensities under the conditions specified in (e) determined by flow cytometry. For (b,d,f), data are expressed as the mean ± standard derivative of three independent experiments. ***p < 0.001 as calculated by the Student’s *t*-test. BF = bright field; FL = fluorescence.

The study was then extended to the other two cell lines, and the results were compared with those of **AsGq-ZnPc** and ZnPc. Figure 3a,b show the confocal images and the flow cytometric data for the cells being incubated with these probes ([ZnPc] = 2 μM) for 4 h. It was found that both **AsGq-4625-ZnPc** and **AsGq-ZnPc** gave strong intracellular fluorescence in MCF-7 and A549 cells. For both cell lines, the difference in intensity was not significant for the two complexes, showing that the effect of 4625 was minimal. In contrast, the fluorescence signal in L929 cells could hardly be observed for both complexes. The results strongly suggested the involvement of nucleolin in the uptake process. For ZnPc, the fluorescence signal was not noticeable for all the three cell lines, which again could be attributed to the strong molecular stacking of this compound in the culture medium. Complexation with an As1411-based G-quadruplex could greatly promote the uptake of this photosensitizer by nucleolin-positive cancer cells.

To reveal whether the two DNA complexes were internalized into the nucleolin-positive cancer cells by receptor-mediated endocytosis, the uptake of **AsGq-4625-ZnPc** against MCF-7 cells was studied upon incubation at two different temperatures (37 and 4 °C) with three different incubation times (1, 2, and 4 h). Upon incubation at 37 °C, strong fluorescence was observed in the cells, and the intensity was found to increase with the incubation time (Figure 3c, left part). In contrast, when the cells were incubated at 4 °C, fluorescence could hardly be observed in the confocal images regardless of the incubation time (Figure 3c, left part). Similar results were obtained by flow cytometry (Figure 3d). The temperature-dependent uptake of this complex suggested that the internalization was mainly through an energy-dependent endocytosis process.

In addition, free G-quadruplex of As1411 (8 μM) was used for preincubation for 30 min before the incubation with **AsGq-4625-ZnPc** ([ZnPc] = 2 μM) for 2 h at 37 °C. As expected, the intracellular fluorescence intensity was reduced significantly upon the preincubation (Figure 3e,f). The results indicated that the G-quadruplex was responsible for the binding with the cell surface markers and could mediate the internalization.

### Subcellular Localization

**Figure 4:**
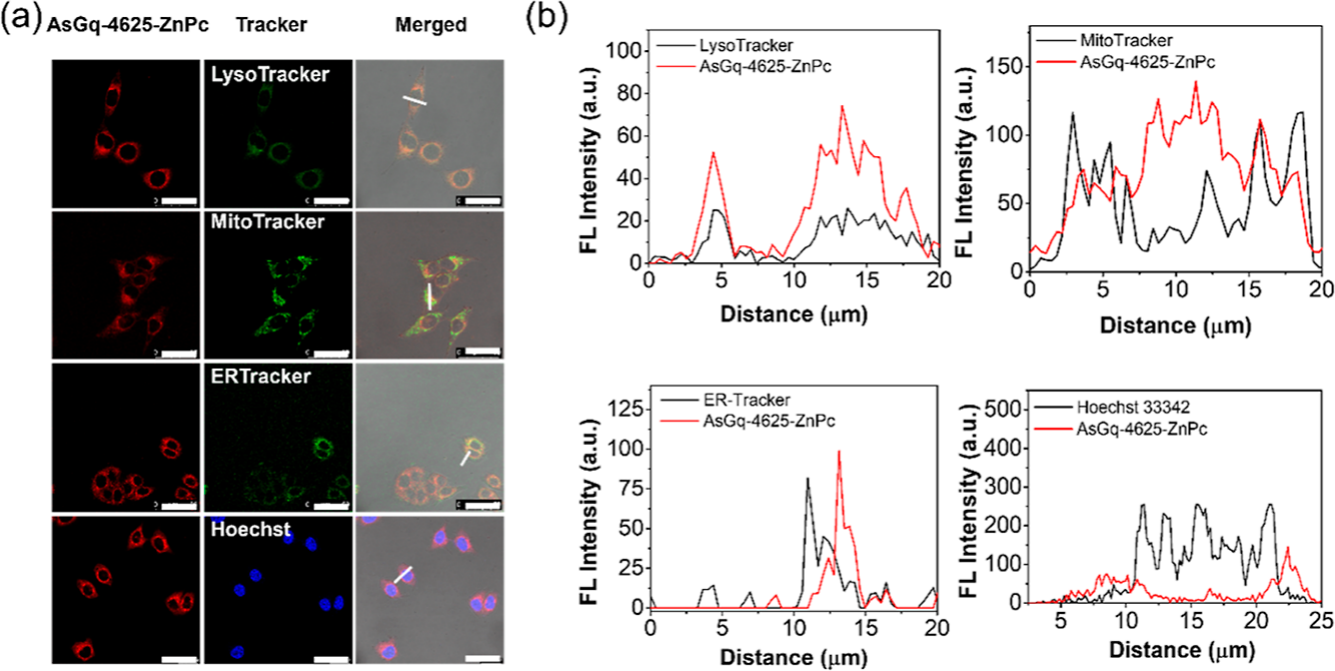
(a) Visualization of the intracellular fluorescence of **AsGq-4625-ZnPc** and various subcellular trackers, as well as their overlapped images. The cells were first incubated with **AsGq-4625-ZnPc** ([ZnPc] = 1 μM) for 4 h at 37 °C. They were then further incubated with LysoTracker Green DND-26 (2 μM), MitoTracker Green FM (0.2 μM), ER-Tracker Green (1 μM), or Hoechst 33342 (1 μM) for 15, 15, 30, and 10 min, respectively, at 37 °C. (b) The fluorescence intensity profiles of **AsGq-4625-ZnPc** and the trackers traced along the white lines in the corresponding images in (a). FL = fluorescence.

The subcellular localization of **AsGq-4625-ZnPc** in MCF-7 cells was further investigated with confocal microscopy. After incubation with **AsGq-4625-ZnPc** ([ZnPc] = 1 μM) for 4 h, the MCF-7 cells were stained with LysoTracker Green DND-26 (2 μM for 15 min), MitoTracker Green FM (0.2 μM for 15 min), ER-Tracker Green (1 μM for 30 min), or Hoechst 33342 (1 μM for 10 min). The intracellular fluorescence of **AsGq-4625-ZnPc** and the trackers generated through selective excitation of the dyes was examined with a confocal microscope. As shown in Figure 4, the fluorescence due to **AsGq-4625-ZnPc** could be overlapped well with that of LysoTracker but not the other three trackers. The results showed that **AsGq-4625-ZnPc** was localized mainly in the lysosomes, which are the last compartments of the endocytic pathway. Hence, escape of the complex from lysosomes is essential in order to achieve antisense therapy.

### Dual Photodynamic and Antisense Therapy

**Figure 5:**
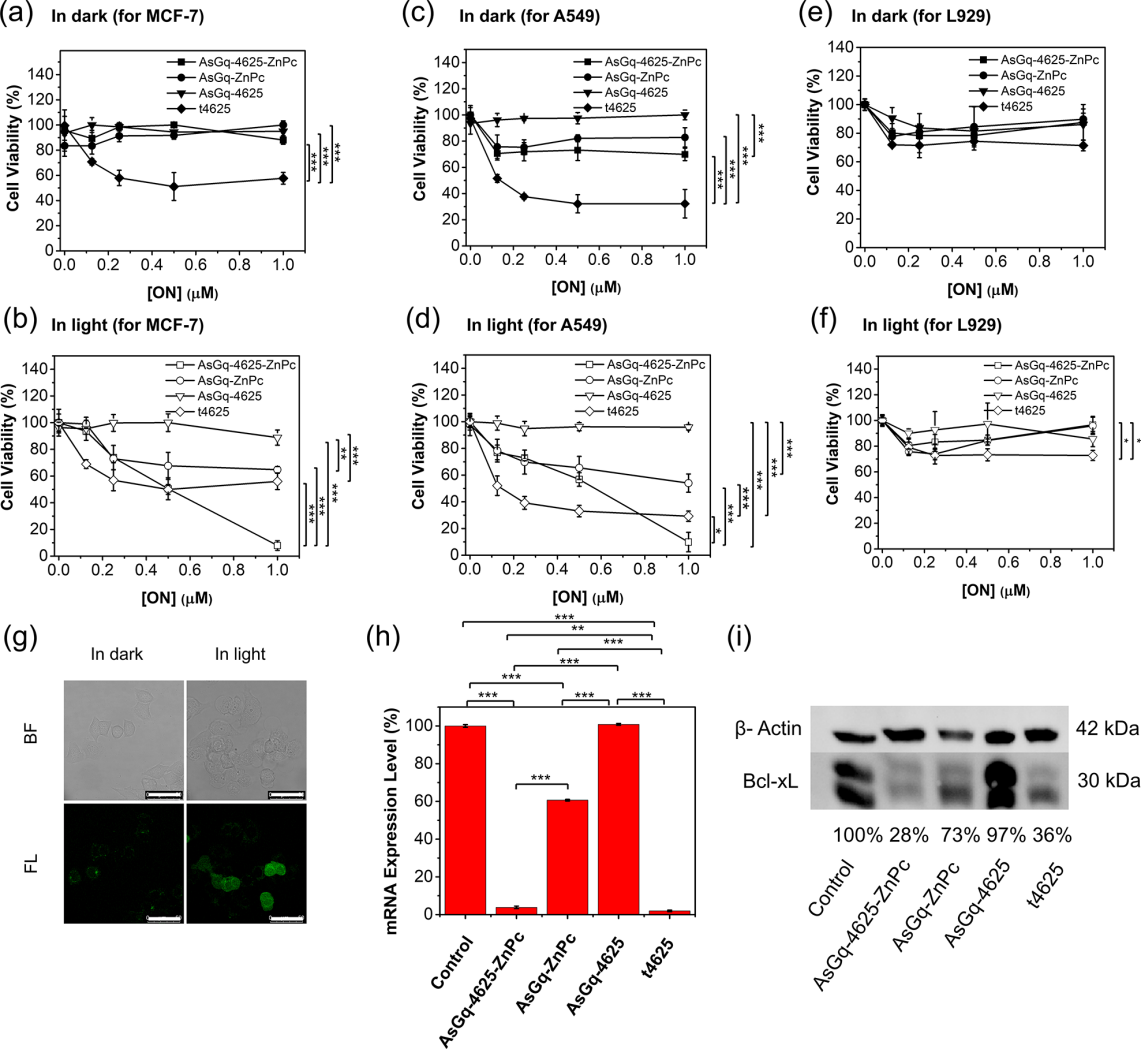
(a,c,e) Dark and (b,d,f) photocytotoxicity of **AsGq-4625-ZnPc**, **AsGq-ZnPc**, **AsGq-4625**, and t4625 against (a,b) MCF-7, (c,d) A549, and (e,f) L929 cells. The cells were incubated with different concentrations of these agents for 4 h, followed by dark or light (λ > 610 nm, 23 mW cm^–2^) treatment for 10 min. Data are reported as the mean ± standard error of the mean of three independent experiments, each performed in quadruplicate. (g) Intracellular ROS generation induced by **AsGq-4625-ZnPc** in MCF-7 cells as indicated by the green fluorescence of DCF. The cells were incubated with **AsGq-4625-ZnPc** ([ZnPc] = 1 μM) for 4 h, followed by incubation with H_2_DCFDA (5 μM) for 30 min. The cells were then subjected to dark or light (λ > 610 nm, 23 mW cm^–2^) treatment for 10 min. BF = bright field; FL = fluorescence. (h) mRNA levels and (i) Western blot of Bcl-xL in MCF-7 cells after the treatment with **AsGq-4625-ZnPc**, **AsGq-ZnPc**, **AsGq-4625**, or t4625 ([ZnPc] = 1 μM, [ON] = 4 μM) for 4 h, followed by light irradiation (λ > 610 nm, 23 mW cm^–2^) for 10 min. *p < 0.05, **p < 0.01, and ***p < 0.001 as calculated by the Student’s *t*-test.

The dark and photocytotoxicity of **AsGq-4625-ZnPc** was then investigated against the three cell lines, and the results were compared with those of **AsGq-ZnPc** and **AsGq-4625**. In addition, 4625 was transfected with lipofectamine 3000, and the resulting t4625 was used as a positive control of antisense therapy. Figure 5 shows the corresponding dose-dependent survival curves determined by MTT assay [MTT = 3-(4,5-dimethylthiazol-2-yl)-2,5-dipheny-2*H*-tetrazolium bromide]. It can be seen in Figure 5a that for MCF-7, all the three AsGq-containing complexes were essentially noncytotoxic in the absence of light irradiation [up to 1 μM ON concentration], while t4625 showed significant cytotoxicity, killing about 40% of the cells at a concentration of 1 μM. In the presence of light irradiation (λ > 610 nm, 23 mW cm^–2^, 13.8 J cm^–2^), the cytotoxicity of **AsGq-4625** and t4625 remained unchanged as expected due to the absence of photosensitizer. For the ZnPc-conjugated **AsGq-ZnPc** and **AsGq-4625-ZnPc**, the cytotoxicity was significantly enhanced. In particular, the latter could eliminate virtually all the cells at 1 μM with a half maximal inhibitory concentration (IC_50_ value) of 0.52 ± 0.02 μM (Figure 5b). The photocytotoxicity of **AsGq-ZnPc** could be attributed to the PDT effect of ZnPc, and the further enhanced photocytotoxicity for **AsGq-4625-ZnPc** was clearly due to the additional cytotoxicity of 4625. It is worth mentioning that ZnPc was noncytotoxic both in the absence and presence of light irradiation (Figure S6). It showed that **AsGq** could effectively enhance the photodynamic activity of ZnPc through disstacking/solubilizing this photosensitizer. As the photocytotoxicity of **AsGq-4625** was negligible (up to 1 μM), the result also showed that the photosensitization of ZnPc in **AsGq-4625-ZnPc** could not only generate ROS for cell killing, but also promote the lysosomal escape of 4625, facilitating the antisense therapy.

The results for A549 cells were generally consistent with those for MCF-7 cells, but these cells seemed to be slightly more susceptible to these agents. As shown in Figure 5c,d, both **AsGq-ZnPc** and **AsGq-4625-ZnPc** were slightly cytotoxic even in the dark, and the cytotoxicity of t4625 was slightly higher for this cell line than for MCF-7 cells, which could eliminate up to 70% of the cells at 1 μM. **AsGq-4625-ZnPc** remained highly potent for this cell line upon light irradiation. The IC_50_ value was determined to be 0.47 ± 0.02 μM. For the nucleolin-negative L929 cells, the dark and photocytotoxicity of all the AsGq-containing complexes was not significant as expected (Figure 5e,f) as a result of the low cellular uptake.

To confirm that ROS were generated upon light irradiation of the ZnPc-complexed G-quadruplexes, 2′,7′-dichlorodihydrofluorescein diacetate (H_2_DCFDA) was used as a ROS probe, which can generate highly emissive 2′,7′-dichlorofluorescein (DCF) upon oxidation by the intracellular ROS.^41^ MCF-7 cells were first incubated with **AsGq-4625-ZnPc** ([ZnPc] = 1 μM) for 4 h, followed by incubation with H_2_DCFDA (5 μM) for 30 min. The cells were then left in the dark or irradiated (λ > 610 nm, 23 mW cm^–2^) for 10 min before being examined with a confocal microscope. As shown in Figure 5g, while the fluorescence of DCF could hardly be observed for the cells receiving the dark treatment, the bright green fluorescence of DCF could clearly be seen upon light irradiation, confirming the generation of ROS under these conditions.

Since 4625 can inhibit the antiapoptotic function of Bcl-xL,^28^ the antisense effect of **AsGq-4625-ZnPc** on the mRNA expression level of this protein was further examined. It has been reported that in MCF-7 cells, Bcl-xL is approximately 10-fold more active than Bcl-2 in suppressing apoptosis induced by doxorubicin.^42^ This cell line was therefore used in this study, and we focused on the effect on Bcl-xL instead of Bcl-2. Again, the results were compared with those of **AsGq-ZnPc**, **AsGq-4625**, and t4625. After incubation with these agents ([ZnPc] = 1 μM, [ON] = 4 μM) for 4 h, followed by light irradiation (λ > 610 nm, 23 mW cm^–2^) for 10 min, the total RNA of the cells was isolated and subjected to real-time reverse transcription polymerase chain reaction (RT-PCR). Glyceraldehyde 3-phosphate dehydrogenase (GAPDH) was used as the internal standard. Serial dilutions of the total RNA of GAPDH and Bcl-xL were quantitative PCR (qPCR)-amplified to obtain the corresponding standard curves (Figure S7). As shown in Figure 5h, **AsGq-4625-ZnPc** exhibited a strong inhibition effect on Bcl-xL mRNA expression reaching as low as 4%, which was comparable with the effect of t4625 (2%). The inhibition ability of **AsGq-ZnPc** was significantly weaker (ca. 60%). Since 4625 was not present in this complex, the weak inhibition effect could be attributed to the PDT effect of ZnPc. For **AsGq-4625**, the effect was negligible. It is likely that without the assistance of photosensitization of ZnPc, the antisense strand could not escape from lysosomes and reach the target mRNA in the cytosol.

Western blot of Bcl-xL protein was then carried out for each of these conditions. As shown in Figure 5i, **AsGq-4625-ZnPc** exhibited the strongest inhibition effect reducing the Bcl-xL protein expression to 28%, which was comparable with that of t4625 (36%). The levels for **AsGq-ZnPc** and **AsGq-4625** were determined to be 73% and 97%, respectively, which were consistent with the results of the RT-qPCR study. These results provided further evidence of the inhibition ability of 4625 in the Bcl-xL mRNA expression level. By comparing the effects of all these DNA, it can be concluded that light can promote the lysosomal escape of 4625 through photosensitization of ZnPc, and both the free 4625 and ZnPc in the cytosol can bind/degrade the Bcl-xL mRNA, hindering the mRNA translation process.

## Conclusions

In summary, we have designed and constructed a self-assembled nucleic acid–based complex for synergistic photodynamic and antisense therapy against cancer. It contains a nucleolin-targeting As1411 sequence as the backbone, which is appended with a short sequence for partial hybridization with the antisense ON 4625, which can inhibit the antiapoptotic function of Bcl-2 and Bcl-xL inducing apoptosis in cancer cells. In the presence of K^+^ ions, this sequence forms a G-quadruplex structure, which can further bind with a ZnPc-based photosensitizer and 4625, forming the target multifunctional therapeutic agent **AsGq-4625-ZnPc** as characterized by various spectroscopic methods and PAGE. Through a series of in vitro experiments using a range of cell lines with different expression levels of nucleolin, we have demonstrated that this DNA complex can be internalized selectively to nucleolin-overexpressed MCF-7 and A549 cells through receptor-mediated endocytosis and is localized mainly in the lysosomes. Upon excitation of the complexed ZnPc with light, ROS can be generated which can induce cytotoxicity by PDT and promote the lysosomal escape of 4625 to cause additional cytotoxicity by antisense therapy. The combined therapeutic effect can eliminate the cancer cells effectively with an IC_50_ value of ca. 0.5 μM. In addition, we have also shown that **AsGq-4625-ZnPc** can effectively inhibit the mRNA and protein expressions of Bcl-xL at the cellular level. The overall results show that this complex with multiple therapeutic components in a single DNA entity serves as a promising tumor-targeting therapeutic agent for synergistic photodynamic and antisense therapy. It is envisaged that this design can be extended to other hybrids with noncancer-related antisense ONs for other clinical applications.

## Experimental Section

### General

The antisense ON and other DNA sequences were purchased from Takara Bio (USA) and Sangon (Shanghai, China), respectively. ONs were diluted in sterile water to a concentration of 400 μM and stored at −20 °C before use. ZnPc was purchased from TCI America (Boston, USA) with purity >95%. All other reagents and solvents were used as received.

Electronic absorption and steady-state fluorescence spectra were taken on a Shimazu UV-1800 UV–vis spectrophotometer and a HORIBA FluoroMax-4 spectrofluorometer, respectively. CD spectra were recorded on a Jasco CD J-150 spectrometer equipped with a Peltier temperature control system. ONs were dissolved in TAMg buffer (0.04 ×) with 40 mM KCl and 2% DMF (v/v). Spectra were acquired in the range of 240–360 nm with a scan speed of 100 nm min^–1^, 1 nm bandwidth, and 1 s integration time over 2 averaged accumulations.

### Preparation of AsGq-4625-ZnPc

The sequence As4625′ was dissolved in a TAMg buffer solution (0.04×) with 40 mM KCl. The solution was annealed at 95 °C for 5 min followed by slow cooling to 4 °C to form the G-quadruplex **AsGq**. The antisense ON 4625 was then added for hybridization to give **AsGq-4625**. Finally, a solution of ZnPc in DMF (0.2 mM, 2 μL) was then mixed with a solution of **AsGq-4625** in TAMg buffer (0.04×) (16 μM, 0.1 mL) with 40 mM KCl to give the target therapeutic agent **AsGq-4625-ZnPc**.

### Determination of Binding Constants

The binding interactions between ZnPc and **AsGq** or **AsGq-4625** in TAMg buffer (0.04×) with 40 mM KCl and 2% DMF (v/v) were studied by fluorescence spectroscopy, and the data were analyzed using a modified Benesi–Hildebrand equation^35^
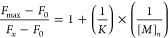
where *F*_0_, *F*_*x*_, and *F*_max_ are the fluorescence intensities of ZnPc at the initial, intermediate, and final (or full complexation) state, respectively, *K* is the binding constant, *M* is the concentration of **AsGq** or **AsGq-4625**, and n is the number of **AsGq** or **AsGq-4625** unit bound to each ZnPc (=4 based on the Job’s plot analysis). The fluorescence intensities of ZnPc (4 μM) in the presence of different concentrations of **AsGq** (0–16 μM) or **AsGq-4625** (0–8 μM) were used for determination of the binding constants.

### Determination of Melting Temperatures

Samples were dissolved in TAMg buffer (0.04×) with 40 mM KCl and 2% DMF (v/v), and the denaturation process was analyzed by monitoring the CD signal at 260 nm in the temperature range of 22–95 °C with a heating rate of 2 °C min^–1^. The melting temperatures were determined using a two-state transition model.

### Study of Biostability

**AsGq-ZnPc** and **AsGq-4625-ZnPc** were dissolved in TAMg buffer (0.04×) with 40 mM KCl and 2% DMF (v/v) at a concentration of 0.24 μM. The solutions were then mixed with 10% FBS in DMEM to give a final concentration of 23.4 ng μL^–1^ DNA, followed by incubation at 37 °C over a period of 24 h. At different time points, an aliquot of the samples was withdrawn for denaturation with 95% formamide at 60 °C for 20 min. The resulting mixtures were then analyzed with 15% denaturing PAGE followed by staining with Shinny green. The band intensity was quantified with ImageJ, and the time-dependent data were fitted according to a first-order exponential decay. In addition, the mixtures were also analyzed by fluorescence spectroscopy. The fluorescence intensity at 685 nm upon excitation at 610 nm was monitored over a period of 24 h.

### Study of Singlet Oxygen Generation

**AsGq-ZnPc**, **AsGq-4625-ZnPc**, and ZnPc were respectively dissolved in TAMg buffer (0.04×) with 40 mM KCl and 2% DMF (v/v) at a concentration of 20 μM with respect to ZnPc. To these mixtures (50 μL), a solution of SOSG in deionized water with 1% dimethyl sulfoxide (DMSO) (50 μM, 1 μL) was added. The resulting mixtures were irradiated with red light coming from a 100 W halogen lamp after passing through a water tank for cooling and a color glass filter (Newport) cut-on at λ = 610 nm. The enhancement of fluorescence intensity of SOSG at λ = 525 nm was monitored with a spectrofluorometer during the irradiation period.

### Cell Lines and Culture Conditions

MCF-7 (ATCC, no. HTB-22) and A549 (ATCC, no. CCL-185) cells were maintained in DMEM (Thermo Fisher Scientific, cat. no. 12100–046) supplemented with FBS (10%, Invitrogen, cat. no. 10270–106) and penicillin–streptomycin (100 unit mL^–1^ and 100 μg mL^–1^, respectively). L929 cells (ATCC, no. CCL-1) were maintained in Eagle’s Minimum Essential Medium (ATCC, cat. no. 30–2003) supplemented with FBS and penicillin–streptomycin (100 unit mL^–1^ and 100 μg mL^–1^, respectively). All the cells were grown at 37 °C under a humidified 5% CO_2_ atmosphere.

### Confocal Fluorescence Microscopic Studies

Approximately 2 × 10^5^ cells in the corresponding medium (2 mL) were seeded on a confocal dish and incubated overnight at 37 °C in a humidified 5% CO_2_ atmosphere. **AsGq-ZnPc** and **AsGq-4625-ZnP**c (0.8 nmol with respect to ZnPc) were dissolved in a mixture of DMF and 40 mM KCl aqueous solution (1:50, v/v, 40 μL) as the stock solutions. For the cellular uptake studies, the cells were incubated with the complexes ([ZnPc] = 2 μM) in the medium (1 mL) for 4 h. As a negative control, the cells were simply incubated with ZnPc (2 μM) in the medium (1 mL) for 4 h. The cells were then rinsed with phosphate-buffered saline (PBS) twice before being examined with a Leica TCS SP8 high-speed confocal microscope equipped with a 638 nm laser. The fluorescence was monitored at 650–750 nm. The images were digitized and analyzed using Leica Application Suite X software.

### Flow Cytometric Studies

Approximately 1 × 10^5^ cells per well in the culture medium were seeded on 12-well plates and incubated overnight at 37 °C under 5% CO_2_. **AsGq-ZnPc** and **AsGq-4625-ZnPc** (0.8 nmol with respect to ZnPc) were dissolved in a mixture of DMF and 40 mM KCl aqueous solution (1:50, v/v, 40 μL) as the stock solutions. The cells, after being rinsed with PBS, were incubated with the complexes ([ZnPc] = 2 μM) in the medium (1 mL) for 4 h. The cells were then rinsed again with PBS and then harvested by 0.25% trypsin–ethylenediaminetetraacetic acid (0.5 mL). The activity of trypsin was quenched with the medium (0.5 mL), and the mixture was centrifuged at 1500 rpm for 3 min at room temperature. The pellet was washed with PBS and then subjected to centrifugation. The cells were then suspended in PBS, and the intracellular fluorescence intensities of the samples were measured with a BD FACSVerse flow cytometer (Becton Dickinson) with 10^4^ cells counted in each sample. The complexes were excited by an argon laser at λ = 640 nm, and the emitted fluorescence was monitored at λ = 720–840 nm. The data collected were analyzed using the BD FACSuite. All experiments were performed in triplicate.

### Study of Subcellular Localization

A stock solution of **AsGq-4625-ZnPc** (20 μM with respect to ZnPc) was prepared as described above, which was then diluted to 1 μM with a serum-free medium. Approximately 2 × 10^5^ MCF-7 cells in DMEM (2 mL) were seeded on a confocal dish and incubated overnight at 37 °C in a humidified 5% CO_2_ atmosphere. The medium was removed, and the cells were rinsed with PBS twice. The cells were then incubated with **AsGq-4625-ZnPc** ([ZnPc] = 1 μM) for 4 h. After being rinsed with PBS twice, the cells were stained with LysoTracker Green DND-26 (Thermo Fisher Scientific, cat. no. L7526) (2 μM for 15 min), MitoTracker Green FM (Thermo Fisher Scientific, cat. no. M7514) (0.2 μM for 15 min), ER-Tracker Green (Thermo Fisher Scientific, cat. no. E34251) (1 μM for 30 min), or Hoechst 33342 (Thermo Fisher Scientific, cat. no. H3570) (1 μM for 10 min) in a serum-free medium at 37 °C. The solutions were then removed, and the cells were rinsed with PBS twice before being examined with a Leica TCS SP8 high-speed confocal microscope equipped with a 488 nm laser and a 638 nm laser. All the trackers were excited at 488 nm and their fluorescence was monitored at 500–570 nm, while the ZnPc moiety was excited at 638 nm and its fluorescence was monitored at 650–750 nm. The images were digitized and analyzed using Leica Application Suite X software.

### Study of Photocytotoxicity

Approximately 2 × 10^4^ cells per well in the corresponding medium (100 μL) were inoculated in 96-well plates and incubated overnight at 37 °C in a humidified 5% CO_2_ atmosphere. Stock solutions of **AsGq-4625-ZnPc**, **AsGq-ZnPc**, and **AsGq-4625** ([ON] = 10 μM) were prepared as described above, which were diluted with a serum-free medium to various concentrations. As a positive control of 4625, t4625 was prepared with the standard protocol using Lipofectamine 3000 reagent (Invitrogen). The cells, after being rinsed with PBS twice, were incubated with 100 μL of different concentrations of these agents for 4 h at 37 °C under 5% CO_2_. The cells were then rinsed with PBS and refed with 100 μL of a serum-containing medium before being irradiated (λ > 610 nm, 23 mW cm^–2^) at ambient temperature for 10 min, giving a total fluence of 13.8 J cm^–2^. Cell viability was determined by means of a colorimetric MTT assay. After irradiation, the cells were incubated at 37 °C under 5% CO_2_ overnight. After incubation, the medium was removed, and the cells were rinsed with PBS twice. A MTT (Sigma) solution in PBS (3 mg mL^–1^, 50 μL) was added to each well followed by incubation for 4 h under the same environment. DMSO (100 μL) was then added to each well. Solutions in all the wells were mixed until homogeneous. The absorbance at 490 nm of each well on the plate was taken by a microplate reader (BioTek Epoch 2 Reader) at ambient temperature. The average absorbance of the blank wells, which did not contain the cells, was subtracted from the readings of the other wells. The cell viability was then determined by the equation
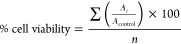
where *A*_*i*_ is the absorbance of the *i*th datum (*i* = 1, 2, . . ., *n*), *A*_control_ is the average absorbance of the control wells in which the drug was absent, and *n* (=4) is the number of data points.

### Determination of Bcl-xL mRNA Expression Levels

After the treatments mentioned above, the total RNA was isolated from the cells using a RNeasy Mini Kit (Qiagen). The extracted RNA was converted to cDNA using SuperScript III First-Strand Synthesis SuperMix (Invitrogen). Real-time amplification was performed according to the Fast SYBR Green Master Mix protocol. Relative quantification of gene expression was determined by a comparative threshold cycle method using GAPDH as an internal standard. For measuring the Bcl-xL mRNA expression level, the corresponding DNA primer pair sequences of the target Bcl-xL were designed with the forward primer in exon 2 and reverse primer in exon 3 using the basic local alignment search tool (BLAST). The sequences of the primers are listed in Table S1. Analysis of the relative gene expression was performed by a StepOnePlus Real-Time PCR System. Relative data were presented in comparison with an untreated control sample. The RT-PCR products were characterized by gel electrophoresis. For this study, 5 ng of each sample was loaded onto a 12% polyacrylamide gel, and the lengths of DNA were compared to the standard DNA ladder. In the mRNA studies, the expected length of the amplicon product of Bcl-xL is 62 bp. The amplicon product was calculated by Primer-BLAST provided by National Institutes of Health, U.S.

### Western Blot Analysis of Bcl-xL Protein Expression

Proteins were extracted from cultured MCF-7 cells by CelLytic M (Sigma), and the quantification was performed by a BCA protein assay (Thermo Scientific) with the use of a microplate reader. For this study, 35 μg of cell lysate was separated by 12% sodium dodecyl sulfate-PAGE gel. The proteins were transferred to a polyvinylidene fluoride membrane with 0.2 μm pore size and an anti β-actin antibody (clone 4C2) supplied by EMD Millipore. The blots were blocked in 3% bovine serum albumin (BSA) (Sigma) with Tris-buffered saline supplemented with 0.1% Tween 20 (TBST) for 1 h. They were then incubated with a Bcl-xL rabbit monoclonal antibody (Cell Signaling Technology) with dilution of 1:1000 in TBST containing 5% BSA at 4 °C overnight. For detection of primary antibodies, the blots were incubated with antirabbit IgG HRP-linked antibody (Cell Signaling Technology) with dilution of 1:1000 in TBST containing 5% BSA at room temperature for 1 h. The blots were stripped and reprobed with a β-actin antibody as the loading control. The immunocomplexes were visualized as enhanced chemiluminescence using an ECL kit. The luminescent bands were detected by a luminescent image analyzer.

## Supporting Information Available

Sequences of the ONs used in this study, CD spectrum of **AsGq**, change in the electronic absorption and fluorescence spectra of ZnPc upon addition of **AsGq**, Job’s plot analysis of the complexation between ZnPc and **AsGq**, denaturing PAGE and fluorescence analyses of **AsGq-ZnPc** and **AsGq-4625-ZnPc** upon FBS digestion, denaturing PAGE analysis of **AsGq-ZnPc** and **AsGq-4625-ZnPc** in DMEM with or without light irradiation, cellular uptake of **AsGq-4625-ZnPc** against MCF-7 cells, dark and photocytotoxicity of ZnPc against MCF-7 cells, as well as the amplification plot and standard curve of RT-qPCR for GAPDH and Bcl-xL (PDF)
Molecular formula strings (CSV)

## Abbreviations Used

Bcl: B cell lymphoma
BF: bright field
BLAST: basic local alignment search tool
BSA: bovine serum albumin
CD: circular dichroism
DCF: 2′,7′-dichlorofluorescein
DLS: dynamic light scattering
DMEM: Dulbecco’s modified Eagle’s medium
DMF: *N*,*N*-dimethylformamide
FBS: fetal bovine serum
DMSO: dimethyl sulfoxide
FL: fluorescence
GAPDH: glyceraldehyde 3-phosphate dehydrogenase
H_2_DCFDA: 2′,7′-dichlorodihydrofluorescein diacetate
IC_50_: half-maximal inhibitory concentration
MTT: 3-(4,5-dimethylthiazol-2-yl)-2,5-dipheny-2*H*-tetrazolium bromide
ON: oligonucleotide
PAGE: polyacrylamide gel electrophoresis
PBS: phosphate-buffered saline
PDI: polydispersity index
PDT: photodynamic therapy
qPCR: quantitative polymerase chain reaction
ROS: reactive oxygen species
RT-PCR: reverse transcription polymerase chain reaction
SOSG: singlet oxygen sensor green
TBST: Tris-buffered saline supplemented with 0.1% Tween 20
TEM: transmission electron microscopy
*T*_m_: melting temperature
ZnPc: zinc(II) phthalocyanine

## References

[ref1] Tamm I.; Dörken B.; Hartmann G. Antisense therapy in oncology: new hope for an old idea?. Lancet 2001, 358, 489–497. 10.1016/S0140-6736(01)05629-X. 11513935

[ref2] Gleave M. E.; Monia B. P. Antisense therapy for cancer. Nat. Rev. Cancer 2005, 5, 468–479. 10.1038/nrc1631. 15905854

[ref3] Taniguchi H.; Suzuki Y.; Imai K.; Adachi Y. Antitumoral RNA-targeted oligonucleotide therapeutics: the third pillar after small molecule inhibitors and antibodies. Cancer Sci. 2022, 113, 2952–2961. 10.1111/cas.15461. 35701833 PMC9459246

[ref4] Plummer R.; Vidal L.; Griffin M.; Lesley M.; de Bono J.; Coulthard S.; Sludden J.; Siu L. L.; Chen E. X.; Oza A. M.; Reid G. K.; McLeod A. R.; Besterman J. M.; Lee C.; Judson I.; Calvert H.; Boddy A. V. Phase I study of MG98, an oligonucleotide antisense inhibitor of human DNA methyltransferase 1, given as a 7-day infusion in patients with advanced solid tumors. Clin. Cancer Res. 2009, 15, 3177–3183. 10.1158/1078-0432.CCR-08-2859. 19383817

[ref5] Khairnar P.; Kolipaka T.; Pandey G.; Phatale V.; Shah S.; Srinivasarao D. A.; Saraf S.; Srivastava S. Nanosponge-mediated oligonucleotide delivery: a cutting-edge technology towards cancer management. J. Drug Delivery Sci. Technol. 2024, 91, 105226 10.1016/j.jddst.2023.105226.

[ref6] Zhao X.; Pan F.; Holt C. M.; Lewis A. L.; Lu J. R. Controlled delivery of antisense oligonucleotides: a brief review of current strategies. Expert Opin. Drug Delivery 2009, 6, 673–686. 10.1517/17425240902992894. 19552611

[ref7] Wahane A.; Waghmode A.; Kapphahn A.; Dhuri K.; Gupta A.; Bahal R. Role of lipid-based and polymer-based non-viral vectors in nucleic acid delivery for next-generation gene therapy. Molecules 2020, 25, 2866 10.3390/molecules25122866. 32580326 PMC7356024

[ref8] Bus T.; Traeger A.; Schubert U. S. The great escape: how cationic polyplexes overcome the endosomal barrier. J. Mater. Chem. B 2018, 6, 6904–6918. 10.1039/C8TB00967H. 32254575

[ref9] Li W.; Wang C.; Zhang Y.; Lu Y. Lipid nanocarrier-based mRNA therapy: challenges and promise for clinical transformation. Small 2024, 20, 2310531 10.1002/smll.202310531. 38287729

[ref10] Safarkhani M.; Ahmadi S.; Ipakchi H.; Saeb M. R.; Makvandi P.; Warkiani M. E.; Rabiee N.; Huh Y. S. Advancements in aptamer-driven DNA nanostructures for precision drug delivery. Adv. Sci. 2024, 11, 2401617 10.1002/advs.202401617. PMC11234471 38713753

[ref11] Espuche B.; Moya S. E.; Calderón M. Nanogels: smart tools to enlarge the therapeutic window of gene therapy. Int. J. Pharm. 2024, 653, 123864 10.1016/j.ijpharm.2024.123864. 38309484

[ref12] Wang M.; Li D.; Zhu J.; Liu J.; Yin Y.; Su Y.; Jin C.; Li J.; Zhang C. Y. Recent advances on two-dimensional material-based nanosystems for gene delivery. APL Mater. 2024, 12, 050601 10.1063/5.0209799.

[ref13] Dong J.; O’Hagan M. P.; Willner I. Switchable and dynamic G-quadruplexes and their applications. Chem. Soc. Rev. 2022, 51, 7631–7661. 10.1039/D2CS00317A. 35975685

[ref14] Gudanis D.; Kaniowski D.; Kulik K.; Baranowski D.; Gdaniec Z.; Nawrot B. Formation of an RNA quadruplex-duplex hybrid in living cells between mRNA of the epidermal growth factor receptor (EGFR) and a G-rich antisense oligoribonucleotide. Cells 2020, 9, 2375 10.3390/cells9112375. 33138194 PMC7692301

[ref15] Zhu J.; Peng L.; Jehan S.; Wang H.; Chen X.; Zhao S.; Zhou W. Activable photodynamic DNA probe with an “AND” logic gate for precision skin cancer therapy. Research 2024, 7, 0295 10.34133/research.0295. 38269029 PMC10807844

[ref16] Wang Q.; Du Y.; Zheng J.; Shi L.; Li T. G-quadruplex-programmed versatile nanorobot combined with chemotherapy and gene therapy for synergistic targeted therapy. Small 2024, 20, 2400267 10.1002/smll.202400267. 38805747

[ref17] Roxo C.; Kotkowiak W.; Pasternak A. G-quadruplex-forming aptamers - characteristics, applications, and perspectives. Molecules 2019, 24, 3781 10.3390/molecules24203781. 31640176 PMC6832456

[ref18] Lopes-Nunes J.; Oliveira P. A.; Cruz C. G-quadruplex-based drug delivery systems for cancer therapy. Pharmaceuticals 2021, 14, 671 10.3390/ph14070671. 34358097 PMC8308530

[ref19] Van den Avont A.; Sharma-Walia N. Anti-nucleolin aptamer AS1411: an advancing therapeutic. Front. Mol. Biosci. 2023, 10, 1217769 10.3389/fmolb.2023.1217769. 37808518 PMC10551449

[ref20] Bøe S. L.; Hovig E. Enhancing nucleic acid delivery by photochemical internalization. Ther. Delivery 2013, 4, 1125–1140. 10.4155/tde.13.78. 24024512

[ref21] Yuan A.; Laing B.; Hu Y.; Ming X. Direct oligonucleotide-photosensitizer conjugates for photochemical delivery of antisense oligonucleotides. Chem. Commun. 2015, 51, 6678–6680. 10.1039/C5CC00573F. PMC4405145 25786195

[ref22] Shi L.; Wu W.; Duan Y.; Xu L.; Xu Y.; Hou L.; Meng X.; Zhu X.; Liu B. Light-induced self-escape of spherical nucleic acid from endo/lysosome for efficient non-cationic gene delivery. Angew. Chem., Int. Ed. 2020, 59, 19168–19174. 10.1002/anie.202006890. 32686235

[ref23] Chen L.; Li G.; Wang X.; Li J.; Zhang Y. Spherical nucleic acids for near-infrared light-responsive self-delivery of small-interfering RNA and antisense oligonucleotide. ACS Nano 2021, 15, 11929–11939. 10.1021/acsnano.1c03072. 34170121

[ref24] Wu Y.; Ding L.; Zheng C.; Li H.; Wu M.; Sun Y.; Liu X.; Zhang X.; Zeng Y. Targeted co-delivery of a photosensitizer and an antisense oligonucleotide based on an activatable hyaluronic acid nanosystem with endogenous oxygen generation for enhanced photodynamic therapy of hypoxic tumors. Acta Biomater. 2022, 153, 419–430. 10.1016/j.actbio.2022.09.025. 36115655

[ref25] Cetin Ersen B.; Goncu B.; Dag A.; Birlik Demirel G. GLUT-targeting phototherapeutic nanoparticles for synergistic triple combination cancer therapy. ACS Appl. Mater. Interfaces 2023, 15, 9080–9098. 10.1021/acsami.2c21180. 36780418

[ref26] Qian Y.; Han Z.; Yang D.; Cai Y.; Jin J.; Yang Z. Metal-organic frameworks facilitate nucleic acids for multimode synergistic therapy of breast cancer. Langmuir 2023, 39, 8205–8214. 10.1021/acs.langmuir.3c00667. 37236267

[ref27] Tarvirdipour S.; Skowicki M.; Maffeis V.; Abdollahi S. N.; Schoenenberger C. A.; Palivan C. G. Peptide nanocarriers co-delivering an antisense oligonucleotide and photosensitizer elicit synergistic cytotoxicity. J. Colloid Interface Sci. 2024, 664, 338–348. 10.1016/j.jcis.2024.03.021. 38479270

[ref28] Zangemeister-Wittke U.; Leech S. H.; Olie R. A.; Simões-Wüst P.; Gautschi O.; Luedke G. H.; Natt F.; Häner R.; Martin P.; Hall J.; Nalin C. M.; Stahel R. A. A novel bispecific antisense oligonucleotide inhibiting both bcl-2 and bcl-xL expression efficiently induces apoptosis in tumor cells. Clin. Cancer Res. 2000, 6, 2547–2555. 10873111

[ref29] Hockenbery D. M.; Oltvai Z. N.; Yin X.-M.; Milliman C. L.; Korsmeyer S. J. Bcl-2 functions in an antioxidant pathway to prevent apoptosis. Cell 1993, 75, 241–251. 10.1016/0092-8674(93)80066-N. 7503812

[ref30] Dailey M. M.; Miller M. C.; Bates P. J.; Lane A. N.; Trent J. O. Resolution and characterization of the structural polymorphism of a single quadruplex-forming sequence. Nucleic Acid Res. 2010, 38, 4877–4888. 10.1093/nar/gkq166. 20348136 PMC2919704

[ref31] Lo P.-C.; Rodríguez-Morgade M. S.; Pandey R. K.; Ng D. K. P.; Torres T.; Dumoulin F. The unique features and promises of phthalocyanines as advanced photosensitisers for photodynamic therapy of cancer. Chem. Soc. Rev. 2020, 49, 1041–1056. 10.1039/C9CS00129H. 31845688

[ref32] Yaku H.; Fujimoto T.; Murashima T.; Miyoshi D.; Sugimoto N. Phthalocyanines: a new class of G-quadruplex-ligands with many potential applications. Chem. Commun. 2012, 48, 6203–6216. 10.1039/c2cc31037f. 22590705

[ref33] Lopes-Nunes J.; Carvalho J.; Figueiredo J.; Ramos C. I. V.; Lourenço L. M. O.; Tomé J. P. C.; Neves M. G. P. M. S.; Mergny J.-L.; Queiroz J. A.; Salgado G. F.; Cruz C. Phthalocyanines for G-quadruplex aptamers binding. Bioorg. Chem. 2020, 100, 103920 10.1016/j.bioorg.2020.103920. 32413624

[ref34] Park H.; Kim J.; Jung S.; Kim W. J. DNA-Au nanomachine equipped with i-motif and G-quadruplex for triple combinatorial anti-tumor therapy. Adv. Funct. Mater. 2018, 28, 1705416 10.1002/adfm.201705416.

[ref35] Sahana A.; Banerjee A.; Lohar S.; Panja S.; Mukhopadhyay S. K.; Matalobos J. S.; Das D. Fluorescence sensing of arsenate at nanomolar level in a greener way: naphthalene based probe for living cell imaging. Chem. Commun. 2013, 49, 7231–7233. 10.1039/c3cc43211d. 23841111

[ref36] Manet I.; Manoli F.; Donzello M. P.; Ercolani C.; Vittori D.; Cellai L.; Masi A.; Monti S. Tetra-2,3-pyrazinoporphyrazines with externally appended pyridine rings. 10. A water-soluble bimetallic (Zn^II^/Pt^II^) porphyrazine hexacation as potential plurimodal agent for cancer therapy: exploring the behavior as ligand of telomeric DNA G-quadruplex structures. Inorg. Chem. 2011, 50, 7403–7411. 10.1021/ic200514z. 21770396

[ref37] Boutorine A. S.; Brault D.; Takasugi M.; Delgado O.; Hélène C. Chlorin-oligonucleotide conjugates: synthesis, properties, and red light-induced photochemical sequence-specific DNA cleavage in duplexes and triplexes. J. Am. Chem. Soc. 1996, 118, 9469–9476. 10.1021/ja960062i.

[ref38] Shieh Y.-A.; Yang S.-J.; Wei M.-F.; Shieh M.-J. Aptamer-based tumor-targeted drug delivery for photodynamic therapy. ACS Nano 2010, 4, 1433–1442. 10.1021/nn901374b. 20166743

[ref39] Yang S.; Ren Z.; Chen M.; Wang Y.; You B.; Chen W.; Qu C.; Liu Y.; Zhang X. Nucleolin-targeting AS1411-aptamer-modified graft polymeric micelle with dual pH/redox sensitivity designed to enhance tumor therapy through the codelivery of doxorubicin/TLR4 siRNA and suppression of invasion. Mol. Pharm. 2018, 15, 314–325. 10.1021/acs.molpharmaceut.7b01093. 29250957

[ref40] Mosafer J.; Teymouri M.; Abnous K.; Tafaghodi M.; Ramezani M. Study and evaluation of nucleolin-targeted delivery of magnetic PLGA-PEG nanospheres loaded with doxorubicin to C6 glioma cells compared with low nucleolin-expressing L929 cells. Mater. Sci. Eng., C 2017, 72, 123–133. 10.1016/j.msec.2016.11.053. 28024568

[ref41] Shen H. M.; Shi C. Y.; Shen Y.; Ong C. N. Detection of elevated reactive oxygen species level in cultured rat hepatocytes treated with aflatoxin B1. Free Radical Biol. Med. 1996, 21, 139–146. 10.1016/0891-5849(96)00019-6. 8818628

[ref42] Fiebig A. A.; Zhu W.; Hollerbach C.; Leber B.; Andrews D. W. Bcl-XL is qualitatively different from and ten times more effective than Bcl-2 when expressed in a breast cancer cell line. BMC Cancer 2006, 6, 213 10.1186/1471-2407-6-213. 16928273 PMC1560389

