# Supplementary material for: Light-Promoted Lysosomal Escape of a Phthalocyanine and Antisense Oligonucleotide-Complexed G-Quadruplex for Dual Photodynamic and Antisense Therapy
Source: ACS Pharmacol Transl Sci. 2024 Sep 25;7(10):3216–27. doi: 10.1021/acsptsci.4c00384 (PMC11475320; doi:10.1021/acsptsci.4c00384)
Supplement: Supplementary file 1 — pt4c00384_si_001.pdf [file pt4c00384_si_001.pdf]

Supporting Information

Light-Promoted Lysosomal Escape of a Phthalocyanine and Antisense Oligonucleotide-Complexed G-Quadruplex for Dual Photodynamic and Antisense Therapy

Dick Yan Tam,* Wendy K. M. Lau, Yosephine Tania Limanto, and Dennis K. P. Ng*

Department of Chemistry, The Chinese University of Hong Kong, Shatin, N.T., Hong Kong, China. E-mail: karentamdy@gmail.com (DYT); dkpn@cuhk.edu.hk (DKPN)

Contents

Table S1 Sequences of the oligonucleotides used in this study.

Figure S1 CD spectrum of **AsGq** in a TAMg buffer solution (0.04×) with 40 mM KCl at ambient temperature.

Figure S2 Change in the (a) electronic absorption and (b) fluorescence (λ_{ex} = 610 nm) spectra of ZnPc (4 μM) upon addition of **AsGq** (up to 40 μM) in TAMg buffer (0.04×) with 40 mM KCl and 2% DMF (v/v). The insets show the variation of the absorbance at 680 nm or fluorescence intensity at 685 nm with the concentration of **AsGq**. (c) Job's plot analysis of the fluorescence data for the titration of ZnPc with **AsGq**. The total concentration of ZnPc and **AsGq** was fixed at 4 μM.

Figure S3 Denaturing PAGE analysis of (a) **AsGq-ZnPc** and (b) **AsGq-4625-ZnPc** after incubation in DMEM with 10% (v/v) FBS at 37 ^{o}C for different periods of time. Figures in the lower part show the change in the area of the band due to **AsGq** or 4625 with the incubation time. Change in fluorescence intensity at 685 nm with the incubation time for (c) **AsGq-ZnPc** and (d) **AsGq-4625-ZnPc** being treated as described above.

Figure S4 15% Denaturing PAGE analysis of **AsGq-ZnPc** (lanes 1 and 2) and **AsGq-4625-ZnPc** (lanes 3 and 4) in DMEM with (lanes 2 and 4) or without (lanes 1 and 3) light irradiation (λ > 610 nm, 23 mW cm^{-2}, 13.8 J cm^{-2}).

Figure S5 (a) Bright field, fluorescence, and the merged confocal images of MCF-7 cells after incubation with various concentrations of **AsGq-4625-ZnPc** ([ZnPc] = 0.5−2 μM) in a serum-free medium for 4 h. Scale bar: 50 μm. (b) Mean intracellular fluorescence intensities of MCF-7 cells under the conditions specified in (a) determined by flow cytometry. (c) Bright field, fluorescence, and the merged confocal images of MCF-7 cells after incubation of **AsGq-4625-ZnPc** ([ZnPc] = 2 μM) in a serum-free medium for 1, 2, and 4 h, respectively. Scale bar: 50 μm. (d) Mean intracellular fluorescence intensities of MCF-7 cells under the conditions specified in (c) determined by flow cytometry.

Figure S6 Dark and photo-cytotoxicity of ZnPc against MCF-7 cells. The cells were incubated with different concentrations of this photosensitizer for 4 h, followed by dark or light ($\lambda > 610$ nm, 23 mW cm^{-2}) treatment for 10 min.

Figure S7 Amplification plot for (a) GAPDH and (b) Bcl-xL over 3–4 orders of magnitude. The total RNA input ranges from 5×10^{-4} to 5×10^{-1} μg in PCR. Standard curve of RT-qPCR for (c) GAPDH and (d) Bcl-xL in MCF-7 cells.

Table S1. Sequences of the oligonucleotides used in this study.

Oligonucleotide	Sequence (5'→3')	ε (at 260 nm) (M^{-1} cm^{-1})
As4625'	GGTGGTGGTGGTTGTGGTGGTGGTGGAA CGGAGGCTGGGAT	401,900
4625	mA*mA*mG*mG*mC*A*T*C*C*C*A*G*C* C*T*mC*mC*mG*mU*mU[a]	185,700
4625'	AACGGAGGCTGGGATGCCTT	192,300
Bcl-xL forward primer	GGTCGCATTGTGGCCTTT	206,000
Bcl-xL reverse primer	TCCTTGTCTACGCTTTCCACG	200,600

[a] "m" denotes 2'-modification with hydroxy group; "*" denotes phosphorothioate modification on the backbone.

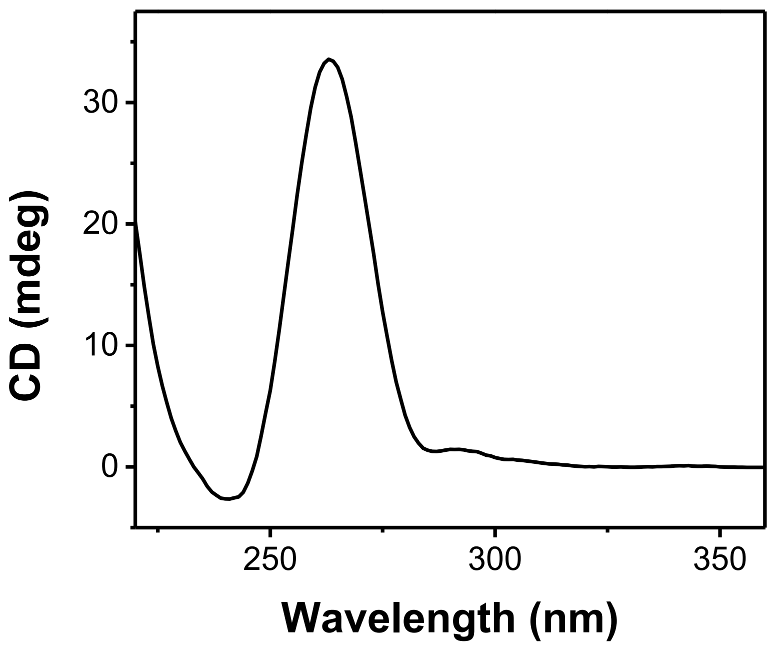

Figure S1. CD spectrum of **AsGq** in a TAMg buffer solution (0.04×) with 40 mM KCl at ambient temperature.

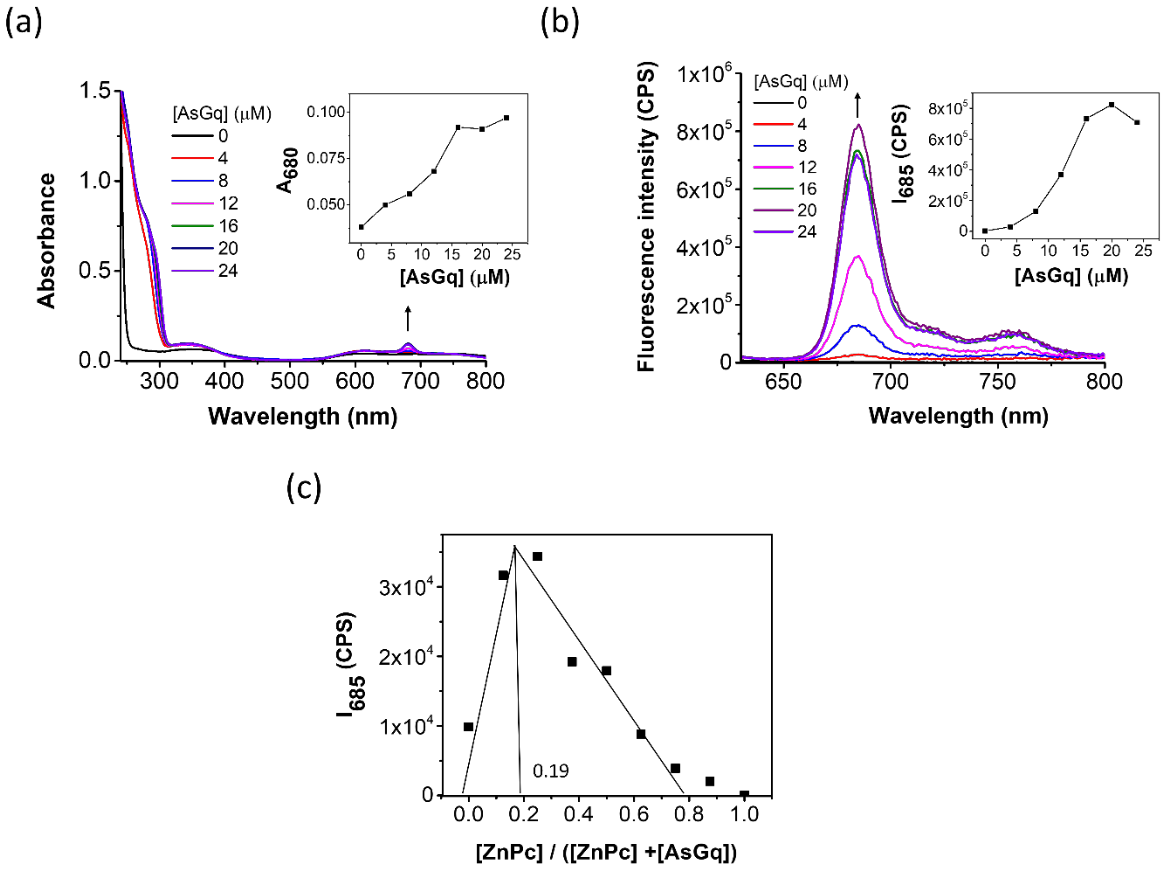

Figure S2. Change in the (a) electronic absorption and (b) fluorescence (λ_{ex} = 610 nm) spectra of ZnPc (4 μM) upon addition of **AsGq** (up to 40 μM) in TAMg buffer (0.04×) with 40 mM KCl and 2% DMF (v/v). The insets show the variation of the absorbance at 680 nm or fluorescence intensity at 685 nm with the concentration of **AsGq**. (c) Job's plot analysis of the fluorescence data for the titration of ZnPc with **AsGq**. The total concentration of ZnPc and **AsGq** was fixed at 4 μM.

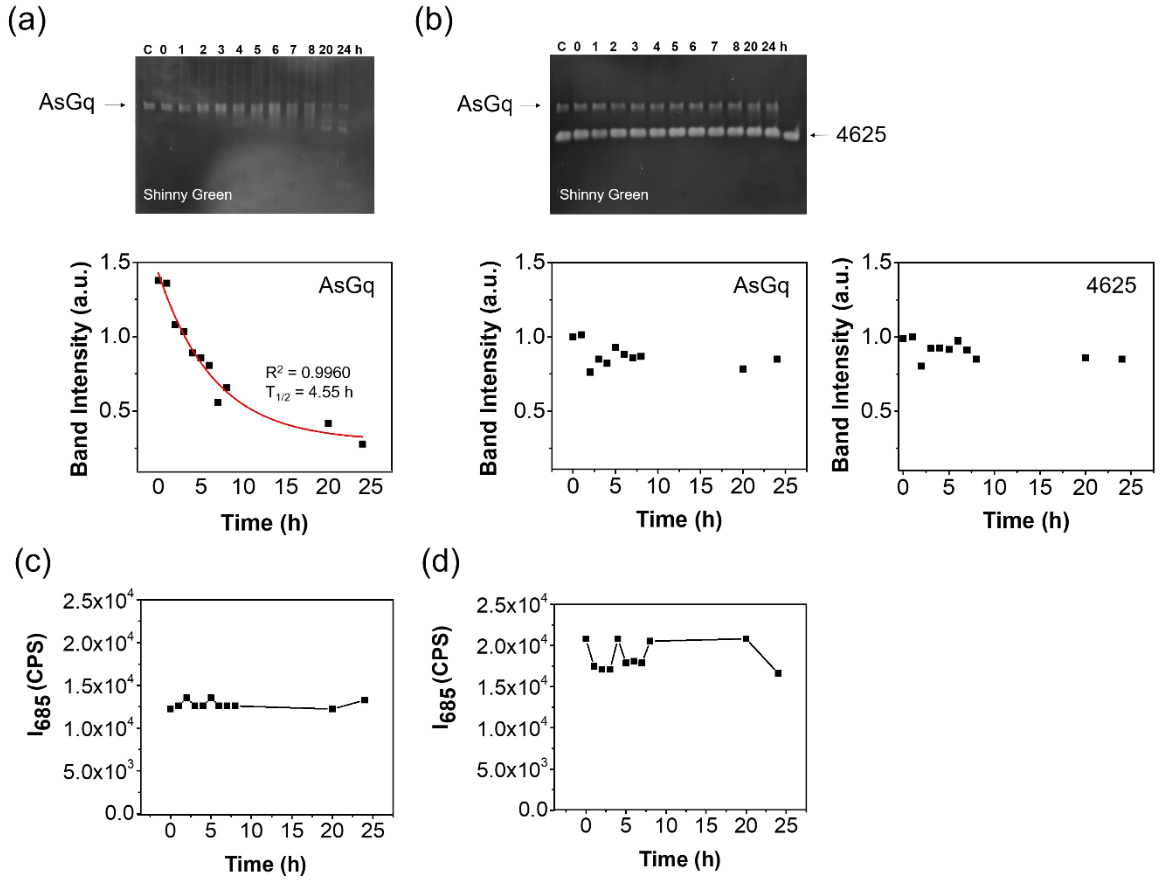

Figure S3. Denaturing PAGE analysis of (a) **AsGq-ZnPc** and (b) **AsGq-4625-ZnPc** after incubation in DMEM with 10% (v/v) FBS at 37 °C for different periods of time. Figures in the lower part show the change in the area of the band due to **AsGq** or 4625 with the incubation time. Change in fluorescence intensity at 685 nm with the incubation time for (c) **AsGq-ZnPc** and (d) **AsGq-4625-ZnPc** being treated as described above.

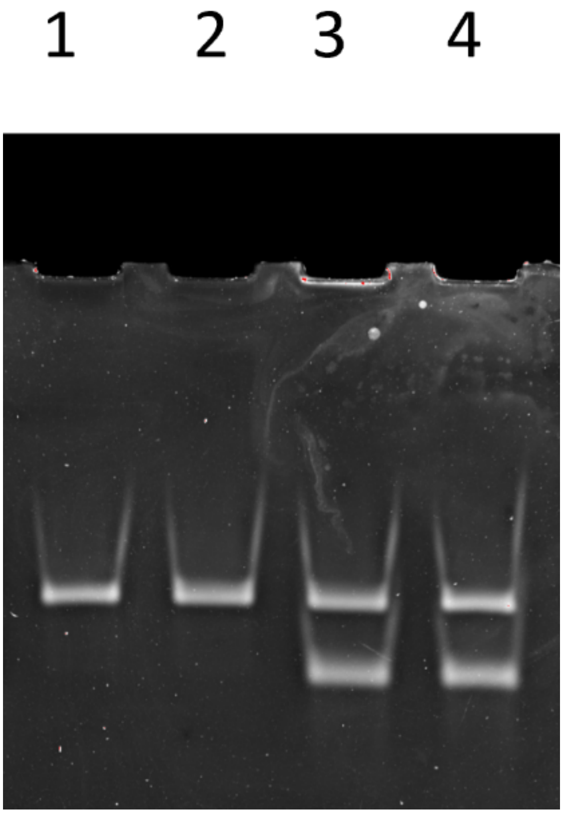

Figure S4. 15% Denaturing PAGE analysis of **AsGq-ZnPc** (lanes 1 and 2) and **AsGq-4625-ZnPc** (lanes 3 and 4) in DMEM with (lanes 2 and 4) or without (lanes 1 and 3) light irradiation (λ > 610 nm, 23 mW cm^{-2}, 13.8 J cm^{-2}).

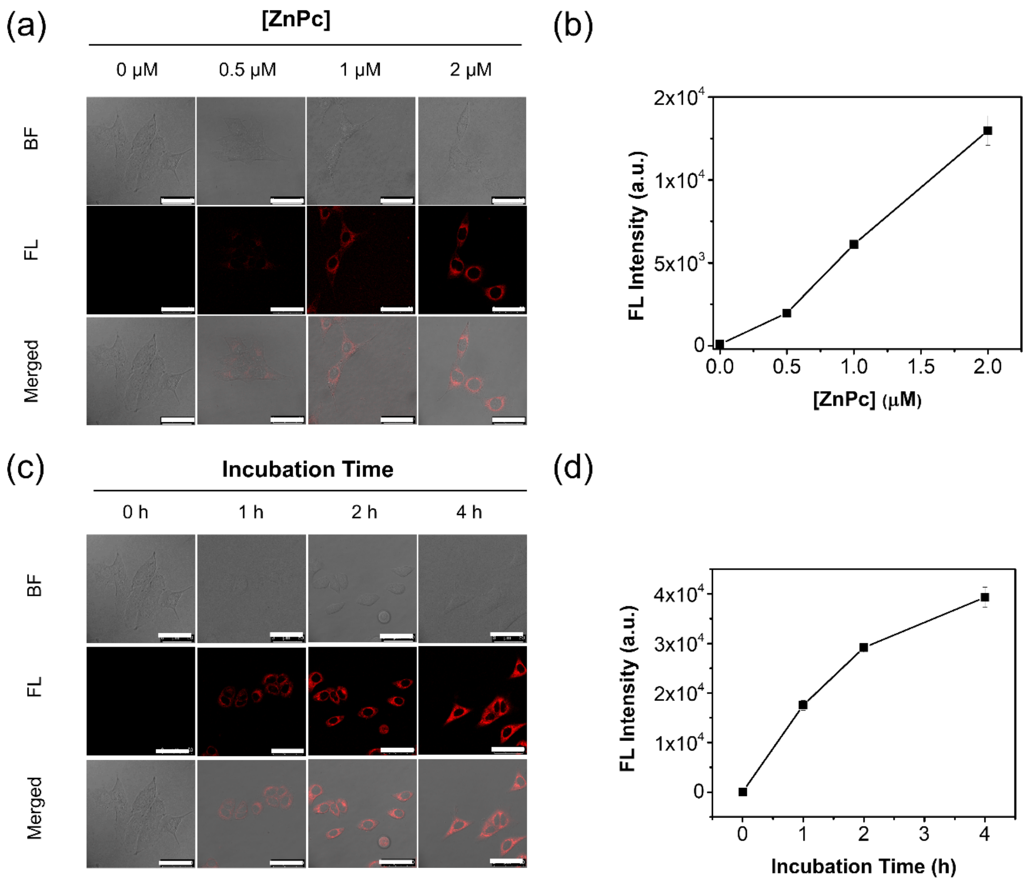

Figure S5. (a) Bright field, fluorescence, and the merged confocal images of MCF-7 cells after incubation with various concentrations of **AsGq-4625-ZnPc** ([ZnPc] = 0.5−2 μM) in a serum-free medium for 4 h. Scale bar: 50 μm. (b) Mean intracellular fluorescence intensities of MCF-7 cells under the conditions specified in (a) determined by flow cytometry. (c) Bright field, fluorescence, and the merged confocal images of MCF-7 cells after incubation of **AsGq-4625-ZnPc** ([ZnPc] = 2 μM) in a serum-free medium for 1, 2, and 4 h, respectively. Scale bar: 50 μm. (d) Mean intracellular fluorescence intensities of MCF-7 cells under the conditions specified in (c) determined by flow cytometry. For (b) and (d), data are expressed as the mean ± standard deviation of three independent experiments. BF = bright field; FL = fluorescence.

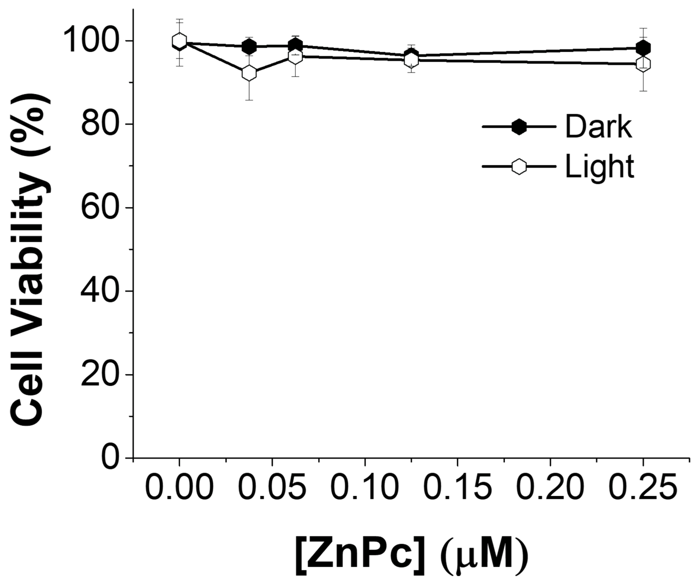

Figure S6. Dark and photo-cytotoxicity of ZnPc against MCF-7 cells. The cells were incubated with different concentrations of this photosensitizer for 4 h, followed by dark or light ($\lambda > 610$ nm, 23 mW cm^{-2}) treatment for 10 min. Data are reported as the mean ± standard error of the mean of three independent experiments, each performed in quadruplicate.

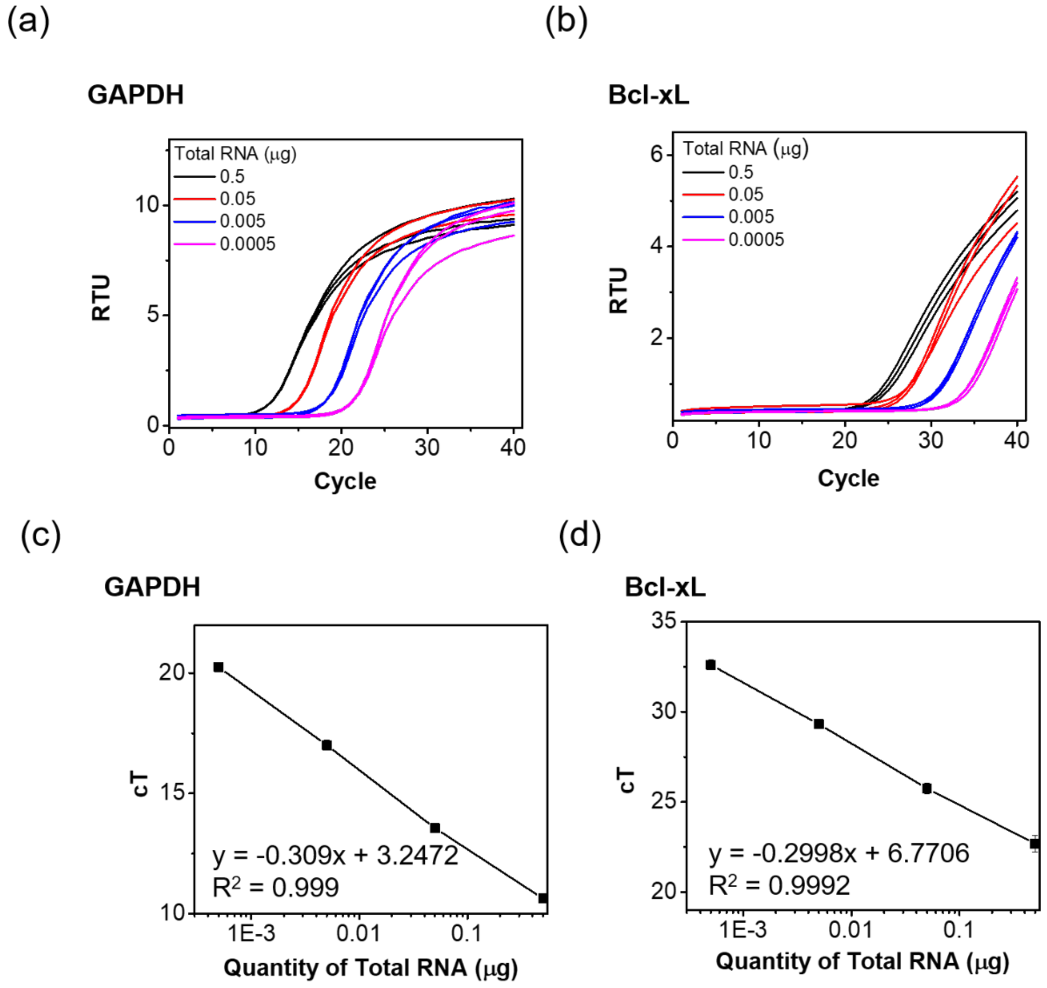

Figure S7. Amplification plot for (a) GAPDH and (b) Bcl-xL over 3–4 orders of magnitude. The total RNA input ranges from 5×10^{-4} to 5×10^{-1} μg in PCR. Standard curve of RT-qPCR for (c) GAPDH and (d) Bcl-xL in MCF-7 cells.
